# Unlocking a Water Coordination Environment in Co-Based Metal–Organic Frameworks for Advanced Nitrate-to-Ammonia Electroreduction

**DOI:** 10.1021/jacs.5c07066

**Published:** 2025-08-06

**Authors:** Pandi Muthukumar, Zakir Ullah, Xia Zhang, Habib Ullah, Yuxiao Liu, Linfeng Li, Shengji Tian, Xianlong Zhou, Savarimuthu Philip Anthony, Yunpeng Zuo, Chade Lv, Xin Wang, Chundong Wang

**Affiliations:** † School of Integrated Circuits, Wuhan National Laboratory for Optoelectronics, 12443 Huazhong University of Science and Technology, Wuhan 430074, P. R. China; ‡ Institut de Ciència de Materials de Barcelona (ICMAB−CSIC), Consejo Superior de Investigaciones Científicas, Campus Universitari de Bellaterra, Cerdanyola del Vallès 08193, Spain; § Department of Engineering, Faculty of Environment Science and Economy, University of Exeter, Exeter EX4 4QF, United Kingdom; ∥ MIIT Key Laboratory of Critical Materials Technology for New Energy Conversion and Storage, School of Chemistry and Chemical Engineering, 47822 Harbin Institute of Technology, Harbin 150001, P. R. China; ⊥ Emergency Center, Hubei Clinical Research Center for Emergency and Resuscitation, 619477 Zhongnan Hospital of Wuhan University, Wuhan 430071, P. R. China; △ School of Chemical & Biotechnology, SASTRA Deemed University, Thanjavur 613401, Tamil Nadu, India; 7 Department of Chemistry, 53025 City University of Hong Kong, Hong Kong 999077, P. R. China

## Abstract

Electrochemical nitrate reduction to ammonia (*e*-NO_3_RR) offers a promising and sustainable alternative to the traditional Haber–Bosch process, enabling decentralized ammonia production under ambient conditions. However, the efficiency of *e*-NO_3_RR is limited by the sluggish reaction kinetics due to the high activation energy barriers, poor mass transport, and the weaker adsorption affinity of the catalyst surface. In this study, we report the design and synthesis of a stable three-dimensional cobalt-based metal–organic framework (HUST-38), constructed from benzene-1,4-dicarboxylate ligand and DABCO, featuring water coordination within its framework. Impressively, the as-prepared HUST-38 delivers a high NH_3_ Faradaic efficiency of 95.7% and a high NH_3_ yield rate of 13.38 mg h^–1^ mg_cat_^–1^ at −0.6 V vs RHE, significantly outperforming the control sample of HUST-39 (3.98 mg h^–1^ mg_cat_^–1^, nonwater coordination) and the mostly reported single-site solid electrocatalysts. Various *in situ* measurements disclose that the labile solvent coordination in HUST-38 promotes water molecule accessibility to the catalytically active metal centers, hence augmenting localized *H enrichment and enhancing NO_3_^–^ reduction. The theoretical calculations further substantiate the essential function of metal coordination microenvironments in modulating the electrocatalytic process, specifically by reducing free energy barriers associated with key reaction intermediates and enhancing the adsorption and desorption kinetics of reactants and products, ultimately leading to improved electrocatalytic activity and efficiency. The present work provides a foundation for the structural design of metal organic frameworks to develop efficient electrocatalysts.

## Graphical abstract

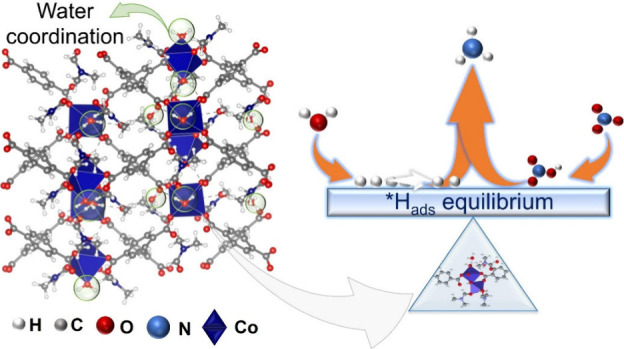

## Introduction

Ammonia (NH_3_) is one of the most widely used industrial chemicals, crucial for nitrogenous fertilizer production and emerging as a promising hydrogen-rich fuel.^1–3^ With a global market of 150 million metric tons annually, NH_3_ is currently produced through the energy-intensive Haber–Bosch process, combining fossil-fuel-derived hydrogen with nitrogen under extreme temperatures and pressures. This process consumes ∼1.4% of the world’s energy and generates ∼1% energy-related carbon dioxide (CO_2_) emissions.^4,5^ Also, extreme nitrate (NO_3_^–^) pollution from overfertilization and industry trash pollutes water systems and harms human health.^6,7^ Electrocatalytic nitrate reduction (*e*-NO_3_RR) offers a feasible, environmentally friendly approach for NH_3_ production, allowing ambient conditions for sustainable energy solutions.^8–13^ A crucial thermodynamic element promoting *e*-NO_3_RR is the relatively low N=O bond dissociation energy of 204 kJ/mol, making NH_3_ synthesis energetically favorable.^14^ However, achieving an effective conversion of NO_3_^–^ to NH_3_ is limited by the complex eight-electron-transfer process, in which it involves the tandem generation and consumption of active hydrogen (*H) from proton reduction or electron transfer reduction. In order to address these challenges, it is crucial to develop electrocatalysts that are incredibly effective. Extensive research has been focused on identifying appropriate electrocatalysts for efficient *e*-NO_3_RR.^15–20^ Interestingly, recent studies have witnessed that *H radicals can serve as an alternative *H source formed from the tensile lattice strain of subsurface O in Ru, catalyzing efficient *e*-NO_3_RR.^21^ The inhibition of water dissociation leads to an insufficient *H generation in *e*-NO_3_RR and a low production rate of NH_3_. In this regard, it is important to design new electrocatalysts that generate a large amount of *H to promote *e*-NO_3_RR for mass production of NH_3_.

Metal–organic frameworks (MOFs), composed of metal ions and organic ligands, have emerged as multifunctional porous materials in electrocatalysis, exploiting their tunable porosity, large surface areas, and flexible structures for tunable activity.^22–25^ However, the bulky organic linkers in MOFs often compromise electrical conductivity and hinder access to active metal sites, resulting in subdued electrocatalytic activity.^26,27^ To overcome this issue, researchers have explored composite MOFs with conducting materials, such as graphene oxide or acetylene black,^28^ and alternative strategies like interlinking metal active centers in coordination polymers, increasing conjugation, and immobilizing transition metal ions (e.g., cobalt and nickel) within 2D conjugated dithiolene ligands.^29,30^ Notably, single-site and molecular catalysts have garnered significant attention due to their optimal active center utilization.^31–34^ Specifically, in the intricate NO_3_^–^ reduction process, the coordination environment and ligand functionality critically impact the MOF electrocatalytic activity. This requires efficient NO_3_^–^ adsorption, hydrogenation facilitated by active *H from H_2_O decomposition, and suppression of the competing hydrogen evolution reaction (HER).

Understanding water molecule reactivity mechanisms is crucial for optimizing catalysts in water electrolysis, enabling sustainable energy technologies including hydrogen production, energy storage, and carbon neutrality.^35–38^ Recent advances in water environment modulation have significantly enhanced kinetics, signifying the importance of subtle variations in water coordination, as exemplified by two Co-MOFs with similar coordination modes exhibiting drastically different electrocatalytic activities.^39^ Rational design strategies have emerged as the key to unlocking improved reactivity, including tailored dinuclear copper complexes synergizing with boric acid buffers and the strategic utilization of “missing linkers” in Co-BDC, revealing their potential as water-coordination sites to improve electrocatalytic activity.^40,41^ These breakthroughs in water environment modulation underscore the importance of rational design in advancing electrocatalysis. Thus, innovative strategies are vital for developing electrocatalysts with water coordination engineering that are optimized for the environmentally friendly *e*-NO_3_RR pathway, favoring ammonia production.

Despite recent advances in *e*-NO_3_RR electrocatalysis, existing MOFs face significant challenges, including compromised accessibility to active metal sites and insufficient *H generation from water decomposition. To overcome these limitations, we leveraged controllable functional groups in precursor modules and strong cobalt­(II) coordination to synthesize two Co-MOFs, HUST-38 and HUST-39 (HUST = Huazhong University of Science and Technology), with distinct coordination microenvironments. Single-crystal X-ray diffraction (SCXRD) reveals multiple coordination water molecular environments in the 3D supramolecular *π–π* stacking structure of HUST-38, whereas HUST-39 lacked water coordination. As expected, HUST-38 demonstrated exceptional NO_3_^–^ electroreduction efficiency, for which it was confirmed that NH_3_ is derived from the reduction of NO_3_^–^ by ^15^N isotope labeling. The integration of experimental results, *in situ* measurements, and theoretical calculations demonstrated that the water coordination environment in HUST-38 allows the generation of an enormous amount of *H from water dissociation, which is subsequently utilized for hydrogenation of nitrogen intermediates, thereby synergistically improving Faradaic efficiency (FE) and NH_3_ yield rate. This study addresses existing gaps in *e*-NO_3_RR electrocatalysis through a systematic investigation of MOFs, ultimately contributing to the development of efficient electrocatalysts for the NO_3_RR.

## Results and Discussion

### Synthesis and Structural Characterizations of HUST-38 and HUST-39

Solvothermal reaction of Co^2+^ with 1,4-benzenedicarboxylic acid (BDC) and 2,5-pyridinedicarboxylic acid (PDC) in the presence of different co-linkers like DABCO and 2,2′-bipyridine produced HUST-38 and HUST-39,^42^ respectively (Figure 1a and 1b). The synthesized single crystals were characterized by SCXRD, and the crystallographic data for the structure were deposited at the Cambridge Crystallographic Data Centre (CCDC) under the deposition numbers 2246025 and 2360130 (Supporting Information provides detailed crystallographic data). Single crystal structural analysis of HUST-38 reveals the presence of two Co metal centers (Co1 and Co2) with different coordination (Figure 1a), in which both Co metal centers displayed octahedral coordination geometry. The carboxylic units of the BDC ligand displayed a monodentate as well as a bidentate mode of coordination with the Co metal center (Figure S1). Co1 and Co2 metal centers are interconnected by the bidentate coordination of two BDC ligands and a water molecule. The Co1 metal center coordinated with the BDC ligand, two dimethylformamide (DMF), and a water molecule, whereas the Co2 metal center coordinated with BDC ligands and a water molecule. Apart from coordinated water and solvent molecules, a DMF and three water molecules are also occupied as lattice solvents in the network structure (Figure S2a). The coupled solvent molecules showed extensive intermolecular H-bonding (Figure S2b). The extended coordination of the BDC ligand enables the formation of 3D porous MOFs with guest molecules occupying the pores (Figure S2a). Although the Co-terephthalic acid MOF with a similar coordination environment has been reported previously, the crystals were unstable upon exposure to air.^43^ A detailed structural comparison between HUST-38 and the previously reported analogue reveals subtle yet critical differences in both unit cell parameters and intermolecular interactions, highlighting that our as-prepared HUST-38 crystal has stable structural frameworks (Figure S3 and Table S1). The Co metal center in HUST-39 also exhibits octahedral coordination geometry (Figure 1b).^42^ Noteworthy, the coordination environment of Co in HUST-39 is completely different from that of HUST-38 (Figure S4a). The Co ion coordinates with bipyridine and a PDC ligand via mono- and bidentate coordination of the pyridine dicarboxylate and bipyridine nitrogen (Figure S4a and S4b), excluding coordination with any volatile solvent. As shown in Figure S4b, HUST-39 presents a 2D network structure, indicating that the two MOFs showcase contrasting coordination environments. More specifically, HUST-38 contains labile solvent molecule coordination, whereas strong ligand coordination is observed alone in HUST-39. The contrasting coordination environments allowed us to explore their role in catalytic performance. The coordination of the labile solvent might make the metal center catalytically active and enhance the electrocatalytic activity.

**Figure 1:**
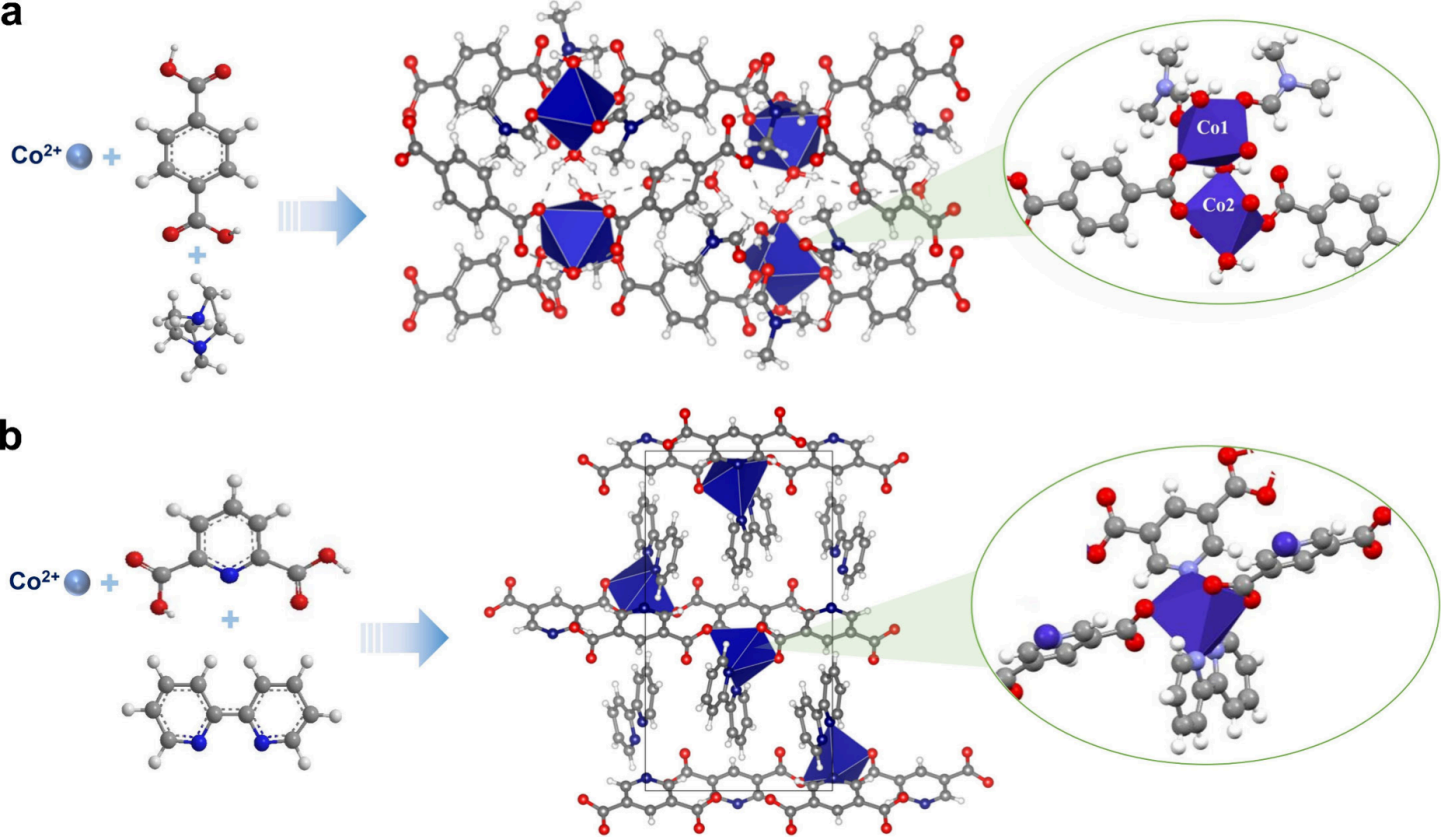
Schematic representation of the synthesis, molecular structure, and coordination network within the crystal lattice of (a) HUST-38 and (b) HUST-39 catalyst. C (gray), H (white), N (sky blue), O (red), and Co (royal blue, polyhedral arrangement).

In accordance with simulated powder X-ray diffraction (PXRD) results, the collected experimental PXRD patterns of HUST-38 and HUST-39 demonstrate great crystallinity and phase purity (Figure S5a and S5b). Thermogravimetric analysis (TGA) shows that lattice water/DMF in HUST-38 can be completely removed below 110 °C, with the structural framework decomposed after 210 °C (Figure S6a). In contrast, HUST-39 was decomposed beyond 210 °C, suggesting that there is no weak/lattice coordination in the framework (Figure S6b). X-ray photoelectron spectroscopy (XPS) further elucidates the cobalt­(II) coordination environment in both MOFs (Figures S7 and S8). Specifically, O 1s core-level spectra of HUST-38 were deconvoluted into three distinct components, Co–O (530.7 eV), C–O (531.9 eV), and Co–O–H (533.1 eV) (Figure S7), whereas HUST-39 exhibited only two deconvoluted peaks (Figure S7), that is, Co–O (530.5 eV) and C–O (531.9 eV), further confirming the water coordination environment in HUST-38.^44,45^ Besides, Co 2p core-level spectra were also analyzed, in which Co 2p_3/2_ (782.2 eV) and Co 2p_1/2_ (798.1 eV) were discerned, signifying that the oxidation state of cobalt in both HUST-38 and HUST-39 is +2 (Figure S8).^45,46^ This result is consistent with the crystallographic data (Supporting Information Notes 1 and 2), which collectively validate the +2 oxidation state of Co.

### Electrochemical Activity and Stability of Nitrate Reduction Reaction

The *e*-NO_3_RR performance of HUST-38 and HUST-39 was evaluated by using a three-electrode H-type cell under ambient conditions. The catalysts were uniformly deposited onto carbon cloth substrates (1 cm^2^) with a mass loading of ca. 0.15 mg cm^–2^. Linear sweep voltammetry (LSV) plots of the samples were recorded, with all potentials referred to the reversible hydrogen electrode (RHE). Noticeably higher current densities were observed in the presence of NO_3_^–^ as compared to the counterpart under NO_3_^–^-free conditions (Figure 2a). A further comparison of the LSV curves reveals that HUST-38 performs with better electrocatalytic activity than HUST-39, as evidenced by a much larger current density (−75.3 mA cm^–2^) and a lower cathodic overpotential at 10 mA cm^–2^ (η_10_ = −0.48 V). It suggests that the favorable water coordination environment in HUST-38 is responsible for this enhanced performance.

**Figure 2:**
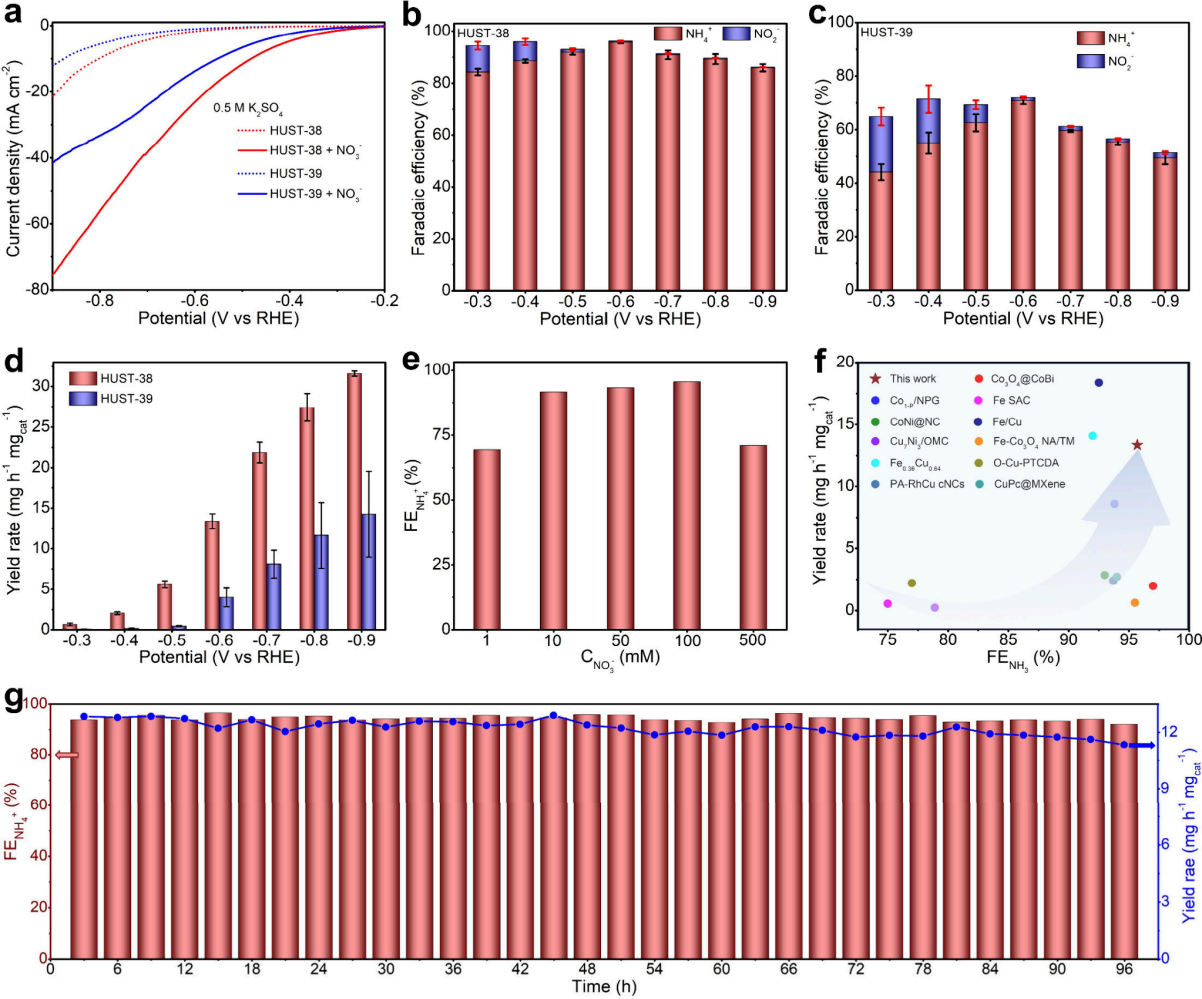
*e*-NO_3_RR performance. (a) LSV curves of HUST-38 and HUST-39 in a 0.5 M K_2_SO_4_ electrolyte and a 0.1 M NO_3_^–^/0.5 M K_2_SO_4_ mixed electrolyte. (b) NH_4_^+^ and NO_2_^–^ FE of HUST-38 at different applied potentials. (c) NH_4_^+^ and NO_2_^–^ FE of HUST-39 at different applied potentials. (d) NH_4_^+^ yield rate of HUST-38 and HUST-39 at different applied potentials. (e) Investigation of FE at various NO_3_^–^ concentrations in a 0.5 M K_2_SO_4_ electrolyte at an applied potential of −0.6 V. (f) Comparison of the *e*-NO_3_RR to NH_3_ performance of HUST-38 with those of other reported electrocatalysts. (g) Chronoamperometric stability test of HUST-38 at −0.6 V (vs RHE) in 0.5 M K_2_SO_4_/0.1 M NO_3_^–^, showing the yield rate and the Faradaic efficiencies for NH_4_^+^ (with electrolyte replacement each 3 h). Catalyst loading for all of the *e*-NO_3_RR tests is 0.15 mg cm^–2^. Error bars denote the standard deviations from at least three independent measurements.

The product selectivity of HUST-38 and HUST-39 was assessed in a K_2_SO_4_/KNO_3_ electrolyte using chronoamperometry at a constant potential for 1 h (Figures S9, S10 and Tables S2, S3). Ultraviolet–visible (UV–vis) spectrophotometry and calibration curves were employed to quantify the concentrations of NH_3_, NO_2_^–^, NO_3_^–^, and H_2_ (Figures S11, S12, S13, S14 and Table S4). HUST-38 and HUST-39 display notable variations in their FE values for the generation of NH_4_^+^ and NO_2_^–^ (Figure 2b and 2c). In more detail, HUST-39 presents a comparatively low FE of 44.1 at −0.3 V vs RHE, and the maximum FE is 70.6% at the cathodic potential of −0.6 V vs RHE (Figure 2c and Table S3). By comparison, it was found that HUST-38 shows remarkable FE values, maintaining high FE over the whole potential range and reaching 95.7% FE at −0.6 V vs RHE (Figure 2b and Table S2). A detailed comparison of ammonia yield rates (YR_NH4_^+^) for HUST-38 and HUST-39 is presented in Figure 2d. With a high YR_NH4_^+^ (HUST-38) of 13.38 mg h^–1^ mg_cat_^–1^ at −0.6 V vs RHE, HUST-38 significantly outperforms HUST-39 by a ratio of around 3.4. This significant improvement highlights the crucial importance of the water coordination environment in HUST-38, generating a large amount of *H and effective NO_3_^–^ reduction to form NH_3_ while suppressing the HER within the specified potential range. Chronoamperometry experiments at −0.6 V further validated the durability of HUST-38’s exceptional performance. Figure S15 demonstrates that the catalyst sustains a consistent yield of YR_NH4_^+^ and FE across time, exhibiting no deterioration following electrolyte exchange at 0.5 and 2 h intervals (Figure S16).

To clarify the origin of NH_3_, specifically whether it arises from NO_3_^–^ reduction or from impurities in the electrolyte and/or air, nuclear magnetic resonance (^1^H NMR) measurement was carried out. For comparison, control experiments were conducted in a 0.5 M K_2_SO_4_ solution without NO_3_^–^ as well (Figure S17). As expected, no NH_4_^+^ was detected throughout the entire potential range. The electrochemical studies were also performed for the bare CC, which shows a lower current density and very low yield rate (0.03 mg h^–1^ cm^–2^), signifying its negligible *e*-NO_3_RR performance (Figure S18). To further prove the advanced performance of our as-synthesized HUST-38 for NO_3_RR and NH_4_^+^ yield, a ^1^H NMR measurement was conducted. As is well-known, isotope labeling coupled ^1^H NMR spectroscopy enables precise tracing of the nitrogen source in NH_3_ production.^47,48^ Prior to the measurement, calibration curves were generated using standard ^14^NH_4_Cl and ^15^NH_4_Cl samples (Figures S19 and S20) to quantify NH_3_ production. Subsequent chronoamperometry experiments were conducted at −0.6 V for 1 h in 0.1 M ^15^NO_3_^–^ and ^14^NO_3_^–^ as a nitrogen source, respectively, with 0.5 M K_2_SO_4_ (Figure S21). Based on the obtained results of ^1^H NMR spectra, distinct spectral patterns for ^15^N ^1^H NMR and ^14^N ^1^H NMR were identified (Figure S22). The ^15^N ^1^H NMR spectra displayed distinct doublet peaks for ^15^NH_4_^+^, while the ^14^N ^1^H NMR spectra showed triplet peaks for ^14^NH_4_^+^ (Figure S22). It was determined that the NH_4_^+^ yield rate and FE at −0.6 V vs RHE were 13.4 mg h^–1^ mg_cat_^–1^ and 96.2% for ^14^NO_3_^–^ and 13.8 mg h^–1^ mg_cat_^–1^ and 96.7% for ^15^NO_3_^–^. The reliability of these results was confirmed by the excellent agreement between the FEs and NH_4_^+^ yields measured by ^1^H NMR and UV–vis spectrophotometry (Figure S23).

The influence of the initial NO_3_^–^ concentrations (*C*_NO_3__^–^) on the *e*-NO_3_RR performance of HUST-38 including FE and yield rate was studied, the results of which were depicted in Figure 2e. The FE stays stable at the 0.01–0.1 M range at −0.6 V, while the FE decreases with the concentration up to 0.5 M due to nitrate/nitrite accumulation. Interestingly, it was noticed that 0.1 M *C*_NO_3__^–^ is the ideal threshold for FE maximization. Figure 2f and Table S5 provide a summary of the important performance measures, showing competitive outcomes of our HUST-38 in comparison to current literature studies reported elsewhere.^15–20,32–34,49,50^ Among the batch conversion experiments using an initial nitrate concentration of 100 μg/mL, HUST-38 delivers 92.7% selectivity toward ammonia production to assess the NO_3_^–^ removal effectiveness (Figure S24). The residual amounts of nitrate (3.6 μg/mL) and nitrite (0.10 μg/mL) after 3 h of electrolysis decreased to values that are below the WHO drinking water recommendations.^51^ The stability of HUST-38 toward *e*-NO_3_RR was evaluated via chronoamperometry measurement. Since few fluctuations for FE and yield rate were discerned over 96 h (Figure 2g), it faithfully validates that HUST-38 possesses robust stability for NH_4_^+^ production, outperforming most of the recently reported MOFs and molecular material-based catalysts (Table S6). After the chronoamperometry stability tests, the aged catalyst was examined by XPS spectroscopy. By comparison of the samples before and after *e*-NO_3_RR, no significant changes were noted, evidencing the robust nature of our as-synthesized MOF (Figure S25). To further investigate the structural stability after *e*-NO_3_RR, ICP-MS analysis, BET measurement, and FT-IR were carried out on the aged HUST-38, which showed retention of intense peaks after *e*-NO_3_RR, further corroborating the structural stability of HUST-38 (Figure S26, Figure S27, Table S7, and Supporting Notes 3 and 4). Additionally, PXRD analysis confirmed the pH-dependent stability of HUST-38 (Figure S28).

### Understanding High-Rate NH_3_ Production on HUST-38

A systematic electrochemical kinetic study was performed to elucidate the fundamental mechanism underlying the *e*-NO_3_RR. The Tafel slopes derived from LSV curves allow us to gain insights into the electroreduction kinetics of the NO_3_RR on HUST-38 and HUST-39. Figure 3a demonstrates that HUST-38 has a Tafel slope (242.2 mV dec^–1^) that is smaller than that of HUST-39 (260.4 mV dec^–1^). The observed difference in Tafel slopes implies that HUST-38 possesses a more favorable kinetic pathway for the *e*-NO_3_RR. Electrochemical impedance spectroscopy (EIS) measurements reveal that HUST-38 exhibits faster charge transfer kinetics compared to HUST-39 under various reaction conditions (Figure S29 and Table S8). To gain deeper insights, additional electrochemical tests were performed to study the electrochemical properties of HUST-38 and HUST-39 and clarify the function of the active site. To determine the electrochemical surface area (ECSA) of the samples, the capacitance current densities were collected from cyclic voltammograms (CVs) recorded in a non-Faradaic region at various scan rates (Figure S30). According to the calculated double-layer capacitance (*C*_dl_) and ECSA values (Figure S31), HUST-38 possesses both higher *C*_dl_ and larger ECSA values than those of HUST-39, which reveals the enhanced active site accessibility of HUST-38 compared to that of HUST-39.

**Figure 3:**
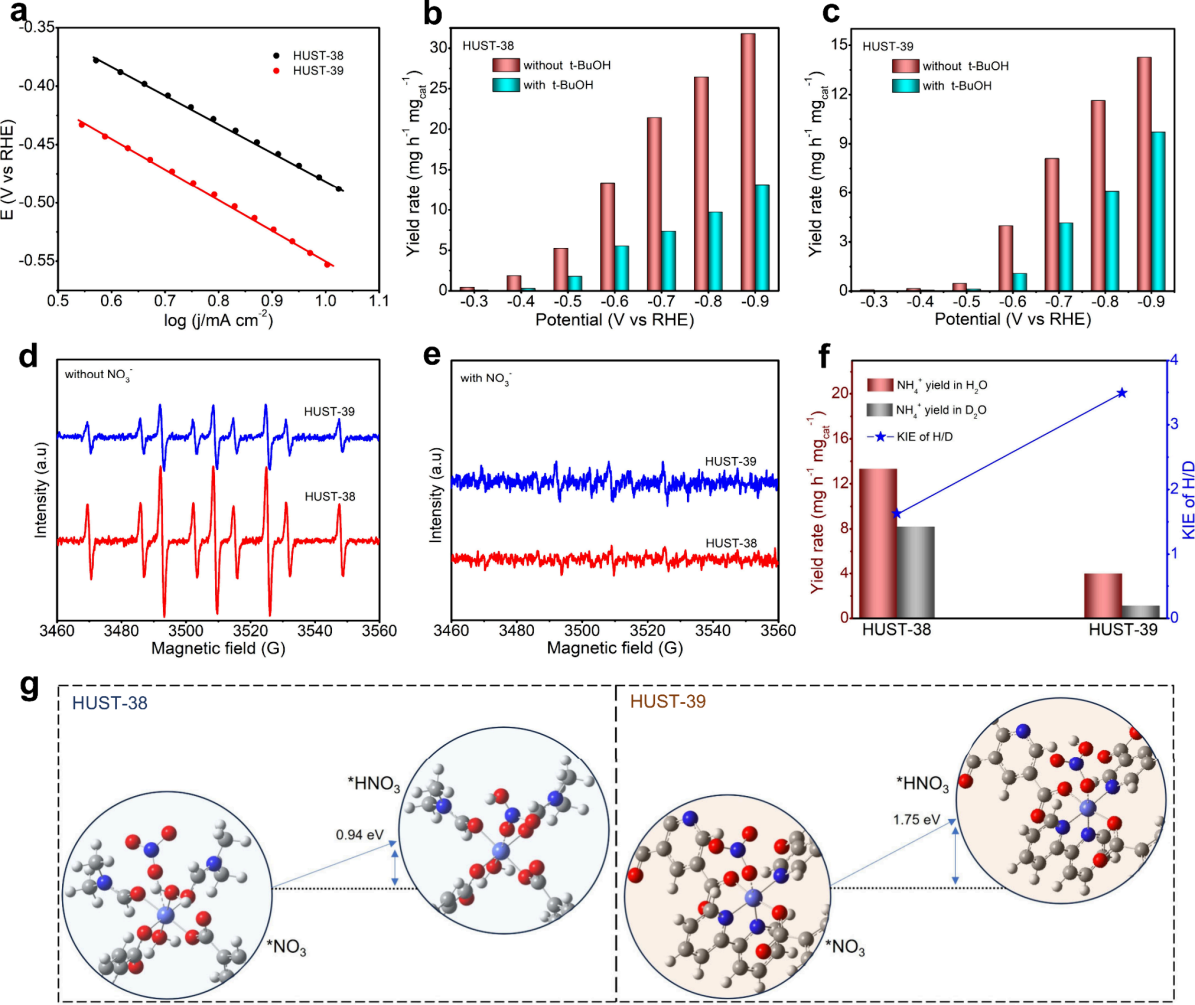
Promoting *e*-NO_3_RR efficiency via active hydrogen-enhanced NH_3_ generation. (a) Tafel plots for HUST-38 and HUST-39 in 0.5 M K_2_SO_4_ with 0.1 M NO_3_^–^, derived from linear sweep voltammetry (LSV) curves recorded at a scan rate of 1 mV/s. (b, c) NH_3_ yield rates of HUST-38 and HUST-39 under a potential range from −0.3 to −0.9 V vs RHE with and without 0.5 M *t*-BuOH quencher. (d, e) ESR spectra of HUST-38 and HUST-39 with/without 0.1 M NO_3_^–^ using DMPO as the radical trapping reagent (each line is collected after 10 min of electrolysis at −0.6 V). (f) Investigation of kinetic isotope effects (KIEs) for hydrogen (H) and deuterium (D) on HUST-38 and HUST-39 catalysts in 0.5 M K_2_SO_4_/0.1 M NO_3_^–^ electrolytes. (g) Calculated free energy profiles for the *NO_3_ to *HNO_3_ transition on HUST-38 and HUST-39 catalysts.

In order to achieve a highly selective *e*-NO_3_RR to NH_3_, the NO_3_RR should follow a sequential process with accelerated NO_3_^–^-to-NO_2_^–^ and NO_2_^–^-to-NH_3_. As such, we assessed the electrocatalytic preferences of both catalysts for *e*-NO_3_RR and *e*-NO_2_RR by comparing the potential differences determined from LSV curves in 0.5 M K_2_SO_4_ with 0.1 M NO_3_^–^ and 0.1 M NO_2_^–^ electrolytes, respectively. As shown in Figure S32, impressively, HUST-38 exhibits a lower onset potential than HUST-39 and depicts a strong preference for both *e*-NO_3_RR and *e*-NO_2_RR. Additionally, HUST-38 displays the smallest potential differences between *e*-NO_3_RR and *e*-NO_2_RR, suggesting that the water coordination in the framework promotes the water adsorption and dissociation, in turn enhancing the *H-rich environment and accelerating both sequential NO_3_^–^-to-NO_2_^–^ and NO_2_^–^-to-NH_3_ reactions. *H is essential to the *e*-NO_3_RR, which proceeds through two distinct steps: deoxygenation and hydrogenation.^52–55^ Figure 3b and 3c show the experimental result using *tert*-butanol (*t*-BuOH) to quench *H radicals, in which lower yield rates for HUST-38 and HUST-39 are observed. It clarifies the crucial role of dynamic *H balance in *e*-NO_3_RR,^53,54^ which emphasizes the vital role of *H in facilitating the production of NH_3_. Furthermore, the presence of *H radicals was directly confirmed by electron spin resonance (ESR) spectroscopy using DMPO (5,5-dimethyl-1-pyrroline-*N*-oxide) as a selective radical trapping agent.^55^ As evidenced by the ESR spectra displaying nine peaks with a hyperfine splitting of DMPO-*H adducts (Figure 3d and 3e), a direct relationship exists between the *H concentration and the DMPO-*H signal intensity, indicating that HUST-38 exhibits a superior water dissociation capacity compared with HUST-39 (Figure 3d). Notably, the DMPO-*H signal intensity for HUST-38 decreased upon the addition of 0.1 M NO_3_^–^, contrasting with that for HUST-39 (Figure 3e). This observation suggests that *H radicals generated by water dissociation on HUST-38 surfaces are efficiently utilized during the hydrogenation of resulting intermediates. The critical role of the *H radical supply demand equilibrium in regulating the *e*-NO_3_RR efficiency is validated, aligning well with the experimental findings.

In light of the fact that the proton transfer kinetics is associated with water dissociation,^56^ the H_2_O/D_2_O experiments were carried out to unveil the kinetic isotope effect (KIE). The significant decrease in the ammonia yield rate when using D_2_O as a solvent underscores the crucial role of hydrogen in enhancing the *e*-NO_3_RR catalytic activity (Figure 3f). Figure 3f shows that the obtained KIE values (H_2_O/D_2_O NH_3_ yield rate ratios) exceeded 1 for both catalysts, confirming the fact that N-intermediate hydrogenations are the rate-limiting steps.^53^ Since the KIE value of HUST-38 is 1.62, the accelerated water dissociation and proton transfer kinetics are achieved, likely due to the lattice water coordination-enabled dynamic water exchange. Different from HUST-38, a high KIE value of 3.49 is yielded for HUST-39, indicating kinetically hindered *H migration from water dissociation. To further elucidate the rate-determining steps (RDS), density functional theory (DFT) calculations were conducted. As shown in Figure 3g, a significantly lower energy barrier for *NO_3_ to *HNO_3_ conversion on HUST-38 (Δ*G* = 0.94 eV) is presented compared to HUST-39 (Δ*G* = 1.75 eV), suggesting the protonation step should be the RDS, being in excellent agreement with experimental *e*-NO_3_RR activities.

### Evaluating the *e*-NO_3_RR Mechanism via *in Situ* Spectroscopy

*In situ* Fourier transmission-infrared spectroscopy (FT-IR) was carried out to clarify the reaction intermediates during the *e*-NO_3_RR. The FT-IR spectra of HUST-38 and HUST-39, obtained at potentials ranging from −0.2 to −0.8 V and open-circuit potential (OCP), are shown in Figure 4a–d. As potential increases, the peak at 1361 cm^–1^ for HUST-38 (Figure 4a) gradually intensifies, suggesting continuous NO_3_^–^ consumption occurred.^57^ The peak at 1514 cm^–1^ is assigned to the stretching vibration of *NO, which is a key intermediate in the reduction of NO_3_^–^.^58^ The peaks at 1233 and 1278 cm^–1^ are attributed to the symmetric and asymmetric stretching vibrations of *NO_2_ intermediates.^58,59^ Furthermore, the peaks at 1105, 1585, 1645, and 1431 cm^–1^ are also represented, being indexed to the hydrogenated intermediates of *NH_3_, *NH, *NH_2_, and *H–N–H, respectively.^58,60–62^ The positive peak at 1456 cm^–1^ confirms NH_4_^+^ generation, implying successive deoxygenation and hydrogenation of NO_3_^–^.^62^ The overlap of the *H_2_O (H–O–H) with *NH peaks (broad band) at 1580–1620 cm^–1^ indicates the presence of hydrogen-bonding interactions between the water molecules and the nitrogen-containing species on the surface of the catalyst.^63^ Figure 4b illustrates significant distinctions of HUST-39 compared to HUST-38, including (i) the lack of the *H_2_O peak and the appearance of new bands at 1160–1190 cm^–1^, which are associated with hydroxylamine intermediates (*NH_2_OH); (ii) the peaks at 1690 and 1740 cm^–1^ corresponding to the on-top adsorbed *NO.^58,62^ To further confirm the reaction intermediates, we conducted *in situ* FT-IR measurements using ^15^N labeling. According to the isotope effect, all the fingerprints of the aforementioned N-containing intermediates were shifted to lower wavenumbers (17–30 cm^–1^), suggesting the formation of *^15^NH_4_^+^ (Figure S33 and Supporting Note 5).^64^ These results faithfully validate the fact that the *e*-NO_3_RR catalyzed to NH_3_ by HUST-38 and HUST-39 was realized via different mechanistic routes involving several intermediates.

**Figure 4:**
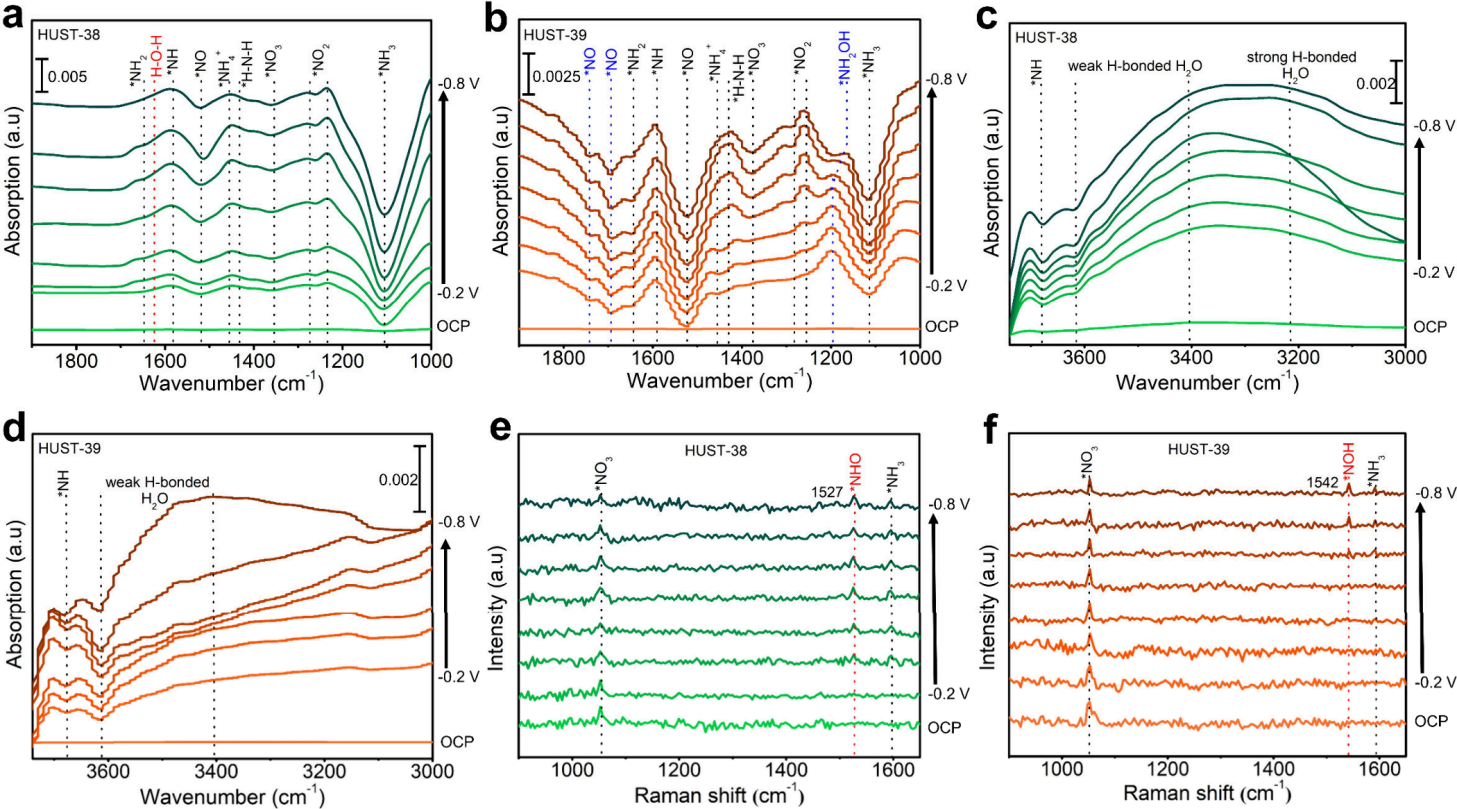
*In situ* spectra to identify the reaction intermediates and reaction pathway were collected in different configurations with 0.5 M K_2_SO_4_/0.1 M NO_3_^–^. (a–d) *In situ* FT-IR spectra for HUST-38 and HUST-39. (e, f) *In situ* Raman spectra for HUST-38 and HUST-39.

The *e*-NO_3_RR kinetics strongly correlates with the fraction of weakly hydrogen-bonded water at the electrochemical interface. In the *in situ* FT-IR spectroscopy of HUST-38, the fingerprint of two distinct water coordination environments is discerned, i.e., weakly H-bonded water molecules (3350–3650 cm^–1^) and strongly H-bonded water molecules (3200–3350 cm^–1^).^65–67^ Thus, it confers that HUST-38 possesses inherent multiple water coordination sites within its framework, which enhances the content of H-bonded H_2_O. Significantly, when the potential increases from −0.2 to −0.6 V, the vibrational band at 3350–3450 cm^–1^ becomes more intense (Figure 4c). This suggests that the catalytic interface has a well-organized configuration of weakly H-bonded H_2_O molecules (>3350 cm^–1^), which facilitates NO_3_^–^ hydrogenation. When the potential is further increased from −0.7 to −0.8 V, the increased content of strong H-bonded H_2_O suppresses NO_3_^–^ reduction, leading to a low FE, as shown in Figure 2b. On the other hand, the absence of a water coordination environment in HUST-39 hinders effective water adsorption, leading to a significantly reduced population of weakly H-bonded water molecules at the catalytic interface (Figure 4d), resulting in poor *e*-NO_3_RR performance. The effective conversion of NO_3_^–^ to NH_3_ for HUST-38 is further supported by the appearance of a distinct N–H stretching peak at 3678 cm^–1^,^65^ which is also observed in HUST-39 (Figure 4c and 4d). Furthermore, *in situ* differential electrochemical mass spectrometry (DEMS) was performed to capture the gaseous products. DEMS results for the HUST-38 electrode showed the *m*/*z* signals of 30, 32, 17, and 18 corresponding to NO, NHOH, NH_3_, and NH_4_^+^, respectively (Figure S34).^68^

*In situ* Raman spectroscopy was also employed to understand the NO_3_^–^–NH_3_ conversion mechanism. As depicted in Figure 4e and 4f, two distinct peaks at 1052 and 1592 cm^–1^ that can be attributed to *NO_3_ and *NH_3_ are observed in both HUST-38 and HUST-39 samples, confirming the successful NO_3_^–^ conversion.^69^ After careful scrutiny, it determines that the peak at 1527 cm^–1^ in the HUST-38 sample is ascribed to *NHO (Figure 4e), whereas the peak at 1542 cm^–1^ in the HUST-39 sample is associated with *NOH intermediates (Figure 4f).^55,70^ Noteworthy, the selection of the N-end route for both catalysts is confirmed by the first proton–electron pair assault on *NO; however, the subsequent transformations of *NO dictate the different reaction pathways, clarify the distinct mechanisms of nitrate reduction on HUST-38 and HUST-39 and emphasize the essential role of *NO intermediates in directing the reaction route. According to the formation of *NO intermediates, our two synthesized samples have two different reaction pathways for *e*-NO_3_RR. Specifically, the *e*-NO_3_RR pathway on HUST-38 proceeds via NO_3_^–^ → *NO_3_ → *HNO_3_ → *NO_2_ → *HNO_2_ → *NO → *NHO → *NHOH → *NH → *NH_2_ → *NH_3_, whereas HUST-39 follows a slightly divergent route: NO_3_^–^ → *NO_3_ → *HNO_3_ → *NO_2_ → *HNO_2_ → *NO → *NOH → *NHOH → *NH_2_OH → *NH_2_ → *NH_3_.

### DFT Mechanistic Study

To verify the *e*-NO_3_RR mechanisms, we calculated the Gibbs free energy with DFT according to the results of the *in situ* FT-IR and Raman measurements. The initial NO_3_^–^ adsorption step is the crucial one for the overall catalytic reaction, which possesses two potential configurations: single oxygen atom adsorption and dual oxygen atom adsorption onto the MOF center. The calculated result discloses that both the configurations have a consistent oxygen adsorption nature, with the binding energy order of HUST-38 (Δ*G* = 0.41 eV) < HUST-39 (Δ*G* = 2.26 eV). The metal–oxygen coordination distances for HUST-38 and HUST-39 are determined to be 1.94 and 2.11 Å, respectively. A comparative analysis of these structures unveils the fact that the enhanced *e*-NO_3_RR kinetics of HUST-38 should be attributed to the weaker proton binding strength of the hydrogen species (ΔG_*H_). Apart from that, the subsequent hydrogenation of adsorbed NO_3_^–^ forming the *HNO_3_ intermediate was also calculated. As depicted in Figure 5a and 5b, the counterpart free energy values are 1.35 eV (HUST-38) and 4.01 eV (HUST-39), respectively.

**Figure 5:**
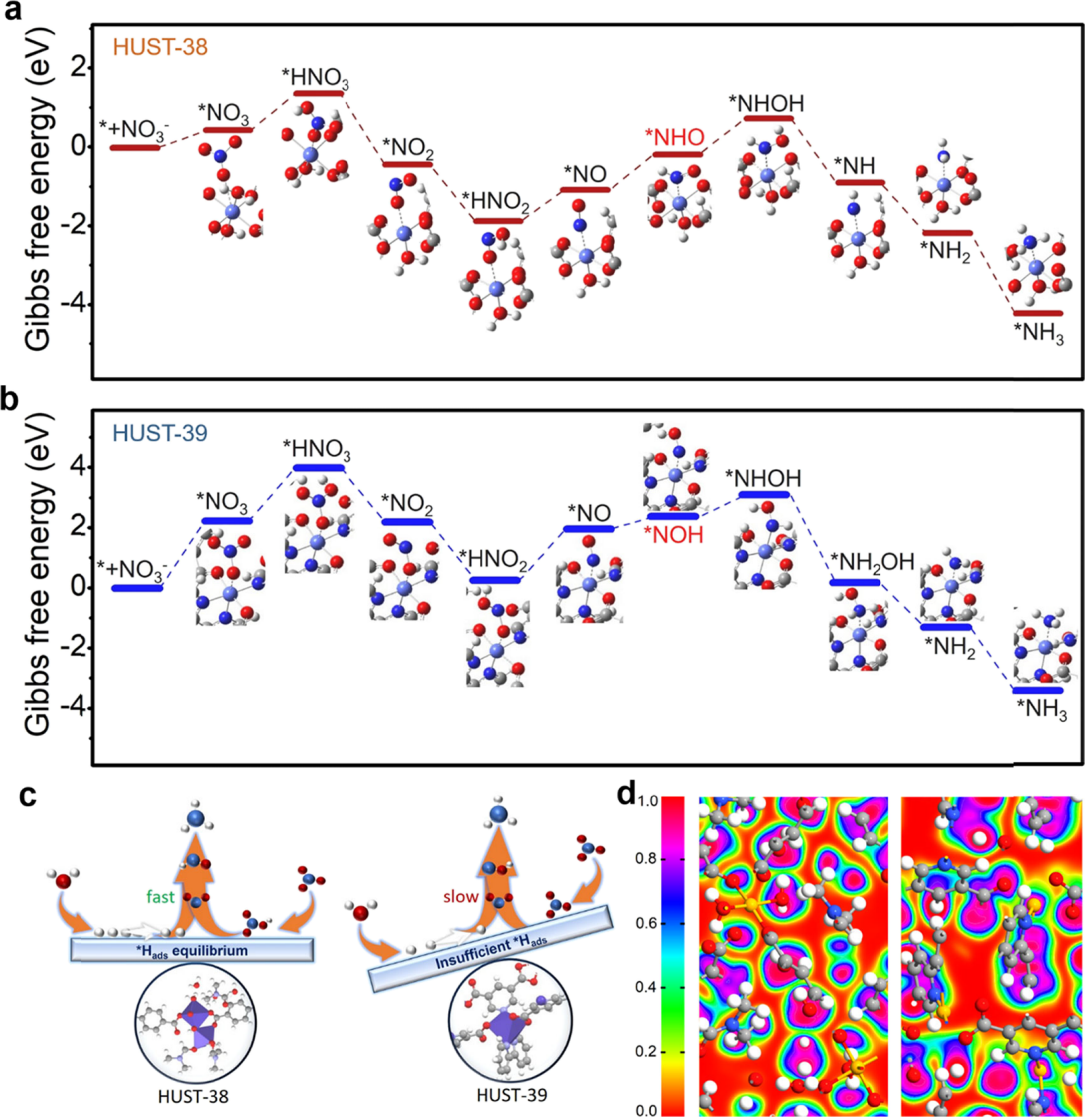
DFT calculations. Gibbs free energy diagrams via the minimum energy pathway and corresponding adsorption configurations of various intermediates generated during the *e*-NO_3_RR for (a) HUST-38 and (b) HUST-39. (c) Schematic illustration of the kinetic reaction mechanism of HUST-38 and HUST-39 in the electroreduction process of NO_3_^–^ to NH_3_. (d) Electron localization function (ELF) analysis of HUST-38 (left side) and HUST-39 (right side).

The DFT results clarify that the formation of *HNO_3_ in the protonation step acts as the potential-determining step (PDS), which is consistent with the experimentally observed KIE values (Figure 3f). The formation of *NO_2_ and *HNO_2_ intermediates is energetically favorable, as indicated by the decrease in their Gibbs free energy. Further calculations of *NO adsorption reveal that the N-end pathway is preferred over the O-end pathway, due to the stronger interaction between the N atom and the metal center. As the energetically favorable pathway, the N-end route is selected for further analysis, highlighting the significance of the exothermic hydrogenation step, where *NO is converted to the *NHO/*NOH intermediate. Both HUST-38 and HUST-39 exhibit a positive charge on the N atom of Co-NO. Especially, back-donation from Co in HUST-38 significantly enhances *NH bond formation, as evidenced by the relatively lower electron charge of 0.06 compared to 0.10 in HUST-39 (Figure S35 and Supporting Note 6). These findings are well aligned with the *in situ* Raman measurements (Figure 4e and 4f). Subsequently, the *NHO/*NOH intermediate undergoes sequential reduction to *NH_3_ via three exothermic H^+^/e^–^ transfer steps at the metal site (Figure S36 and Figure 5c).

The electron localization function (ELF) analysis (Figure 5d) presents the water coordination behaviors in HUST-38, where water molecules share electron density with the adjacent atoms, forming a unique coordination environment. This coordination environment enables the efficient dissociation of water molecules, generating hydrogen radicals (*H). The formation of *H is crucial for catalyzing the *e*-NO_3_RR, as it provides the necessary hydrogen source for the nitrate reduction. In contrast, HUST-39 lacks inherent water coordination sites within its structural framework, hindering the dissociation of water molecules and subsequent *H formation. The simulated work function values corroborate our experimental findings, revealing that HUST-38 possesses a lower work function (3.71 eV) compared to HUST-39 (3.87 eV). This disparity highlights the superior electron transfer efficiency of HUST-38, making it a promising highly efficient electrocatalyst.

### Zinc-Nitrate Battery

Inspired by the outstanding *e*-NO_3_RR performance of HUST-38, we configured a homemade zinc-nitrate (Zn-NO_3_^–^) battery using HUST-38 as the cathode and a Zn plate as the anode in a flow cell device. The cathode and anode were separated by a Nafion 117 membrane. The battery setup delivered a stable open-circuit voltage (OCV) of 1.417 V vs Zn/Zn^2+^ for 2 h (Figure S37a). The discharge polarization and power density curves revealed a peak current density of 15 mA cm^–2^ at 0.298 V (Figure S37b). HUST-38 achieved a power density of 4.42 mW cm^–2^, surpassing most recently reported Zn-NO_3_^–^ batteries (Figure S37b and Table S9). Furthermore, the HUST-38-based Zn-NO_3_^–^ battery discharges at different current densities, revealing steady and stable current responses (Figure S37c), indicating excellent rate stability of the Zn-NO_3_^–^ battery. The HUST-38-based Zn-NO_3_^–^ battery delivered a NH_3_ yield of 1.18 mg h^–1^ cm^–2^ and an FE of 79.2% at 16 mA cm^–2^ (Figure S37d). Additionally, we evaluate the flow cell electrolyzer configuration that effectively converts NO_3_^–^ to NH_3_ (Figure S38 and Supporting Note 7). Notably, the minimal potential difference after *iR* compensation in the flow cell primarily stems from improved electrode kinetics and efficient mass transport, outperforming the H-cell configurations (Figure S39). These results highlight the promising potential of HUST-38 in enhancing the performance of Zn-NO_3_^–^ batteries for both electricity generation and NH_3_ production.

## Conclusions

In summary, we have designed and synthesized two Co-MOFs, HUST-38 and HUST-39, with distinct coordination configurations. Notably, HUST-38 exhibits a unique 3D network structure featuring water coordination within its structural frameworks. Different from HUST-38, HUST-39 has a 2D network structure with 2,2′-bipyridine and PDC ligand coordination. Impressively, HUST-38 displays a high FE of 95.7%, high ammonia yield rate of 13.38 mg h^–1^ mg_cat_^–1^, and better stability over 96 h at −0.6 V vs RHE. Electrochemical testing together with *in situ* measurements evidence that water coordination in the structural framework favors the formation of a large amount of *H intermediates, thereby promoting the electrocatalytic reduction of NO_3_^–^ to NH_3_. The theoretical calculations uncover the origin of the markedly improved *e*-NO_3_RR performance in HUST-38, attributed to water coordination as well, which enables efficient water dissociation and dynamic water exchange within the MOF structure. This study provides a design for stable MOFs and highlights the importance of coordination regulation for efficient electrocatalysis.

## Supplementary Material



## Notes

Materials and methods; characterization; NO_3_RR measurements; product analysis; calculation of ammonia yield rate and FE; computational methodology; supplementary data, figures, and tables; supporting references (PDF)
