# Supplementary material for: Unlocking a Water Coordination Environment in Co-Based Metal–Organic Frameworks for Advanced Nitrate-to-Ammonia Electroreduction
Source: J Am Chem Soc. 2025 Aug 6;147(33):29949–60. doi: 10.1021/jacs.5c07066 (PMC12755198; doi:10.1021/jacs.5c07066)
Supplement: Supplementary file 1 [file ja5c07066_si_001.pdf]

Unlocking Water Coordination Environment in Co-based Metal-Organic Frameworks for Advanced Nitrate-to-Ammonia Electroreduction

Pandi Muthukumar[1†], Zakir Ullah[2†], Xia Zhang[1], Habib Ullah[3], Yuxiao Liu[1], Linfeng Li[1], Shengji Tian[4], Xianlong Zhou[5], Savarimuthu Philip Anthony[6], Yunpeng Zuo[7], Chade Lv[4], Xin Wang[7*], Chundong Wang[1*]

[1]School of Integrated Circuits, Wuhan National Laboratory for Optoelectronics, Huazhong University of Science and Technology, Wuhan, 430074, P. R. China

[2]Institut de Ciència de Materials de Barcelona (ICMAB–CSIC), Consejo Superior de Investigaciones Científicas, Campus Universitari de Bellaterra, Cerdanyola del Vallès, 08193, Spain

[3]Department of Engineering, Faculty of Environment Science and Economy, University of Exeter, Exeter EX4 4QF, United Kingdom

[4]MIIT Key Laboratory of Critical Materials Technology for New Energy Conversion and Storage, School of Chemistry and Chemical Engineering, Harbin Institute of Technology, Harbin 150001, P.R. China

[5]Emergency Center, Hubei Clinical Research Center for Emergency and Resuscitation, Zhongnan Hospital of Wuhan University, Wuhan 430071, P. R. China

[6]School of Chemical & Biotechnology, SASTRA Deemed University, Thanjavur 613401, Tamil Nadu, India

[7]Department of Chemistry, City University of Hong Kong, Hong Kong 999077, P. R. China.

†These authors contributed equally to this work

Corresponding authors: Email: wang.xin@cityu.edu.hk; apcdwang@hust.edu.cn

Experimental Section

Materials synthesis

Synthesis of HUST-38

A novel cobalt-based metal-organic framework (HUST-38) was synthesized via hydrothermal method by dissolving cobalt(II) nitrate hexahydrate (0.1 mM), 1,4-benzenedicarboxylic acid (0.1 mM), and DABCO (0.12 mM) in 10 mL of N,N-dimethylformamide (DMF). The mixture was stirred for 30 minutes at room temperature before being transferred to a Teflon-lined stainless steel hydrothermal reactor, sealed, and heated at 100 °C for 48 hours. The solution was cooled to room temperature and transferred into a beaker. Pink coloured crystals were formed after three months.

Synthesis of HUST-39

HUST-39 was hydrothermally synthesized using a mixture of cobalt(II) nitrate hexahydrate (3 mM), 2,5-pyridinedicarboxylic acid (3 mM), and 2,2'-bipyridine (3.3 mM) in 15 mL of N,N-dimethylformamide (DMF). Following 30 minutes of stirring at ambient temperature, the solution was transferred to a Teflon-lined stainless-steel reactor, sealed, and heated at 120 °C for 36 hours. Upon cooling, the resulting pink crystals were collected, washed with DMF to eliminate impurities, and dried under vacuum, yielding a highly crystalline MOF.

Catalyst characterization

The synthesized material's phase purity and crystallographic structure were investigated using Powder X-ray diffractometry (PXRD) with a Philips X'pert Pro instrument and a Cu Kα X-ray source. The chemical valence states of the samples were determined using Kratos AXIS Ultra DLD-600W X-ray photoelectron spectroscopy (XPS) equipped with a micro focused monochromatic Al Kα X-ray source(1486.6 eV). Single-crystal X-ray diffraction analysis was performed using synchrotron radiation (λ = 0.62998 Å) on an ADSC Quantum-210 detector at the 2D SMC beamline with a silicon (111) double crystal monochromator (DCM). Single crystal data deposited at the Cambridge Crystallographic Data Centre (CCDC number: 2246025 and 2360130). Additionally, thermogravimetric analysis (TGA) was conducted on a PerkinElmer TGA-8000, and surface chemical composition was analyzed using X-ray photoelectron spectroscopy (XPS) on a Thermo-Fisher Scientific K-Alpha instrument. The X-ray photoelectron spectroscopy (XPS) peaks were fitted using XPS peak fitting software. The binding energy ranges from 526 to 538 eV for oxygen and 778 to 812 eV for cobalt. The fitting

parameters, including peak positions and intensities, were optimized to achieve the best fit. The fitting results are presented in the manuscript in detail. The Bruker EMX plus instrument was utilized to perform electron spin resonance (ESR) spectroscopy analysis.

Electrochemical NO_3RR measurements

All electrochemical tests were performed on a CHI 760 workstation using an H-type cell separated by a Nafion 117 membrane at 25 °C. Prior to measurements, the membrane underwent pretreatment: boiling in 3% H_2O_2 for 1 h, rinsing with deionized water, boiling in deionized water for 2 h, and then in 0.5 M H_2SO4 for 1 h, followed by repeated rinsing with deionized water. A homogeneous ink was prepared by ultrasonically dispersing 3.0 mg of electrocatalyst in a mixture of 45 mL deionized water, 0.5 mL ethanol, and 50 μL Nafion. Then, 30 μL of the ink was drop-cast onto pre-treated carbon cloth (1 cm^2), yielding a catalyst loading of 0.15 mg cm^{-2}. The solvent was allowed to evaporate naturally, forming a compact film used as the working electrode.

Nitrate reduction reaction (NO_3RR) experiments were conducted in a three-electrode setup in an H-type cell, utilizing Ag/AgCl (saturated KCl) as the reference electrode and platinum foil as the counter electrode, with potentials converted to the reversible hydrogen electrode (RHE) scale ($E_{RHE} = E_{Ag/AgCl} + 0.198 + 0.0591pH$). The electrolyte consisted of 0.5 M K_2SO_4 with 0.1 M NO_3^- (KNO_3), and all experiments were performed under an ultrahigh-purity Ar gas (99.99%) atmosphere. Prior to electrocatalytic testing, linear sweep voltammetry (LSV) curves were recorded at 5 mV s^{-1} from 0 to -1.0 V until steady-state polarization curves were achieved. Subsequent potentiostatic tests were conducted from -0.3 to -0.9 V vs. RHE (intervals of -0.1 V) for 1 hour to measure ammonia faradaic efficiencies and yield rates. The electrocatalytic active surface area (ECSA) was calculated by analyzing the double-layer capacitance (C_{dl}) extracted from cyclic voltammograms (CVs) measured at scan rates of 20-100 mV/s within the non-Faradaic potential region. The ECSA was then determined using the equation ECSA = C_{dl}/Cs, where Cs represents the specific capacitance (0.04 mF).

Determination of the concentration of N-containing species

Determination of ammonia: The concentration of produced ammonia (NH_3) was spectrophotometrically quantified using a indophenol blue method.[1,2] Briefly, an aliquot of electrolyte was taken out from the electrolytic cell and diluted to within the detection range. A 2 mL sample of the diluted electrolyte was then mixed with 2 mL of a solution containing 1 M NaOH, 5 wt% salicylic acid, and 5 wt% sodium citrate. Subsequently, 1 mL of 0.05 M NaClO

and 0.2 mL of 1.0 wt% $C_5FeN_6Na_2O$ (sodium nitroferricyanide) were added. After 2 hours at room temperature, the absorption spectrum was measured using a UV-vis spectrophotometer. The formation of indophenol blue was determined using the absorbance at a wavelength of 655 nm. A calibration curve was constructed using standard ammonium chloride solutions to correlate concentration and absorbance.

Determination of Nitrite: Nitrite (NO_2^-) concentrations were spectrophotometrically quantified using a colorimetric assay.[3] A color reagent was prepared by dissolving 0.2 g N-(1-naphthyl)ethylenediamine dihydrochloride and 4 g p-aminobenzenesulfonamide in 50 mL deionized water, adding 10 mL phosphoric acid (ρ = 1.685 g mL^{-1}), and mixing thoroughly. For analysis, electrolyte samples from the electrolytic cell were diluted to within the detection range. Then, 5 mL of the diluted sample were mixed with 0.1 mL of color reagent. After 20 minutes at room temperature, the absorption spectrum was measured using a UV-vis spectrophotometer, and the absorbance was recorded at 540 nm. A series of standard potassium nitrite solutions were used to obtain the concentration–absorbance curve by the same procedure.

Determination of Nitrate: Nitrate (NO_3^-) concentrations were spectrophotometrically quantified by adding 0.2 mL of 1 M HCl and 0.02 mL of 0.8 wt% sulfamic acid solution to the diluted sample (2 mL).[4] Absorbance values were recorded at 220 nm and 275 nm. The final absorbance was calculated as $A = A_{220nm} - A_{275nm}$, correcting for potential interferences. A series of standard potassium nitrate solutions were used to obtain the concentration–absorbance curve by the same processes.

Calculation of the Faradaic efficiency (FE) and Yield rate (YR)

$$FE_{NH_4^+} = (8F \times c_{NH_4^+} \times V) / (M_{NH_4^+} \times Q)$$

$$YR_{NH_4^+} = (c_{NH_4^+} \times V) / (t \times M)$$

where $c_{NH_4^+}$ is the mass concentration of NH_4^+ (aq), V is the volume of electrolyte in the cathode compartment (45 mL), $M_{NH_4^+}$ is the molar mass of NH_4^+, t is the electrolysis time (1 h), M is the mass loading of the catalyst (0.15 mg cm^{-2}), F is the Faradaic constant (96485 C mol^{-1}), Q is the total charge passing the electrode.

^{1}H nuclear magnetic resonance (^{1}H NMR)measurements and ^{15}N isotope-labelling experiment

To complement UV-vis results, **^{1}H** NMR spectra were acquired on an 600 MHz SB Liquid Bruker Avance NMR spectrometer. The electrolyte pH was adjusted to weakly acidic using 4 M H_2SO_4. Maleic acid ($C_4H_4O_4$, 50 ppm) served as an external standard for calibrating the

NH_4^+ standard curve via peak area ratio analysis. Additionally, isotopic labeling experiments employing potassium nitrate-^{15}N ($K^{15}NO_3$) were conducted to elucidate the nitrogen source of NH_3. These experiments followed the same protocol as before, with the exception of replacing the N-source with $K^{15}NO_3$, and were performed at -0.6 V vs. RHE and the electrolyte were analysed and quantified.

In-situ/Operando FT-IR and Raman spectroscopy

In-situ Fourier transform infrared (FT-IR) spectroscopy was conducted using a TENSOR II FT-IR spectrophotometer synchronized with a CHI 760 electrochemical workstation. The array electrodes served as the working electrode, with Ag/AgCl and graphite rod employed as reference and counter electrodes, respectively. FT-IR spectra were acquired at a resolution of approximately 30 s per spectrum, concurrently with chronoamperometric measurements, as the potential was swept from open circuit potential to -0.8 V. In-situ Raman measurements (λ = 532 nm, resolution = 2 cm^{-1}) were performed using a Raman microscope coupled with a CHI 760 electrochemical workstation, scanning potentials from -0.2 to -0.8 V (V vs RHE). A custom-designed Teflon cell with a quartz window served as the reactor. The working electrode was submerged in the electrolyte with varied configurations, maintaining a perpendicular orientation to the laser beam. The in-situ Raman spectra were recorded in a chronoamperometric mode with the potential held for 3 min under each fixed potential.

Differential electrochemical mass spectrometry (DEMS) measurements

For the DEMS measurement, 0.5 M K_2SO_4 electrolyte with 0.1 M NO_3^- was kept flowing into a specially designed electrochemical cell through a peristaltic pump. Ar was constantly bubbled into the electrolyte before and during the DEMS measurements. Carbon paper coated with HUST-38 electrocatalyst, a Pt foil, and Ag/AgCl electrode were used as the working electrode, counter electrode, and reference electrode, respectively. Linear sweep voltammetry technology was employed from 0 V to -0.9 V vs. RHE at a scan rate of 10 mV s^{-1} until the baseline was stable. Thereafter, the corresponding mass signals appeared. After the electrochemical test was over and the mass signal returned to the baseline, the next cycle was started. After three cycles, the experiment was ended.

Quasi in-situ ESR tests

Electrochemical measurements were performed using a three-electrode setup, consisting of a carbon cloth working electrode (1 cm^2, loaded with 0.15 mg catalyst), a platinum foil counter electrode, and an Ag/AgCl reference electrode. Chronoamperometry was conducted at a constant potential of -0.6 V vs. reversible hydrogen electrode (RHE) for 5 min with 5,5-dimethyl-1-pyrroline-N-oxide (DMPO) to trap the generated radicals or intermediates, and the resulting solution was subjected to electron spin resonance (ESR) analysis.

Computational Methodology

Solid-state Density Functional Theory (DFT) simulations were conducted using the QuantumATK software, employing the Generalized Gradient Approximation (GGA) functional for electronic structure calculations.[5] The crystal structures of HUST-38 and HUST-39 were directly taken from our experimental crystal structure data. HUST-38 exhibits monoclinic symmetry with space group $P2_1$ and lattice parameters of a = 9.007 Å, b = 17.492 Å, and c = 10.486 Å, with angles $\alpha = \gamma = 90°$ and $\beta = 99.74°$. In contrast, HUST-39 has orthorhombic symmetry, space group $P2_1$, and lattice parameters of a = 9.116 Å, b = 18.170 Å, and c = 9.993 Å, with $\alpha = \beta = \gamma = 90°$. The HUST-38 and HUST-39 models contain 176 and 144 atoms, respectively. We applied the Linear Combination of Atomic Orbitals (LCAO) approach for analyzing Co, N, C, H, and O atoms. The Hartwigsen-Goedecker-Hutter (HGH) pseudopotential was used to optimize computational efficiency, with calculations performed using a basis set of 4 in the LCAO method. The Electron Localization Function (ELF) were analyzed to gain insight into the electronic structure and catalytic potential of these materials.

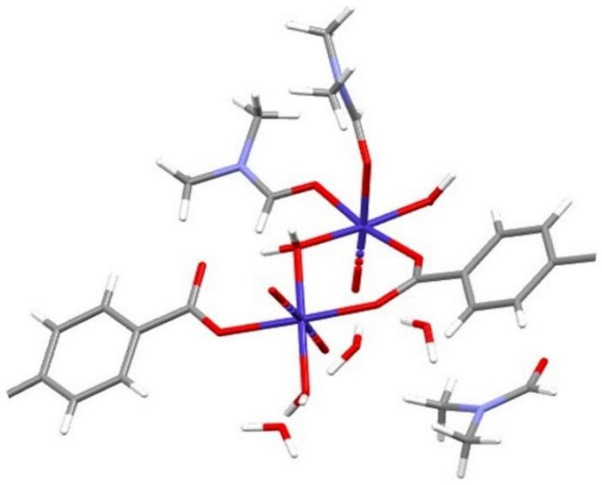

Figure S1. Asymmetric units of HUST-38. C (grey), H (white), N (light blue), O (red) and Co (dark blue).

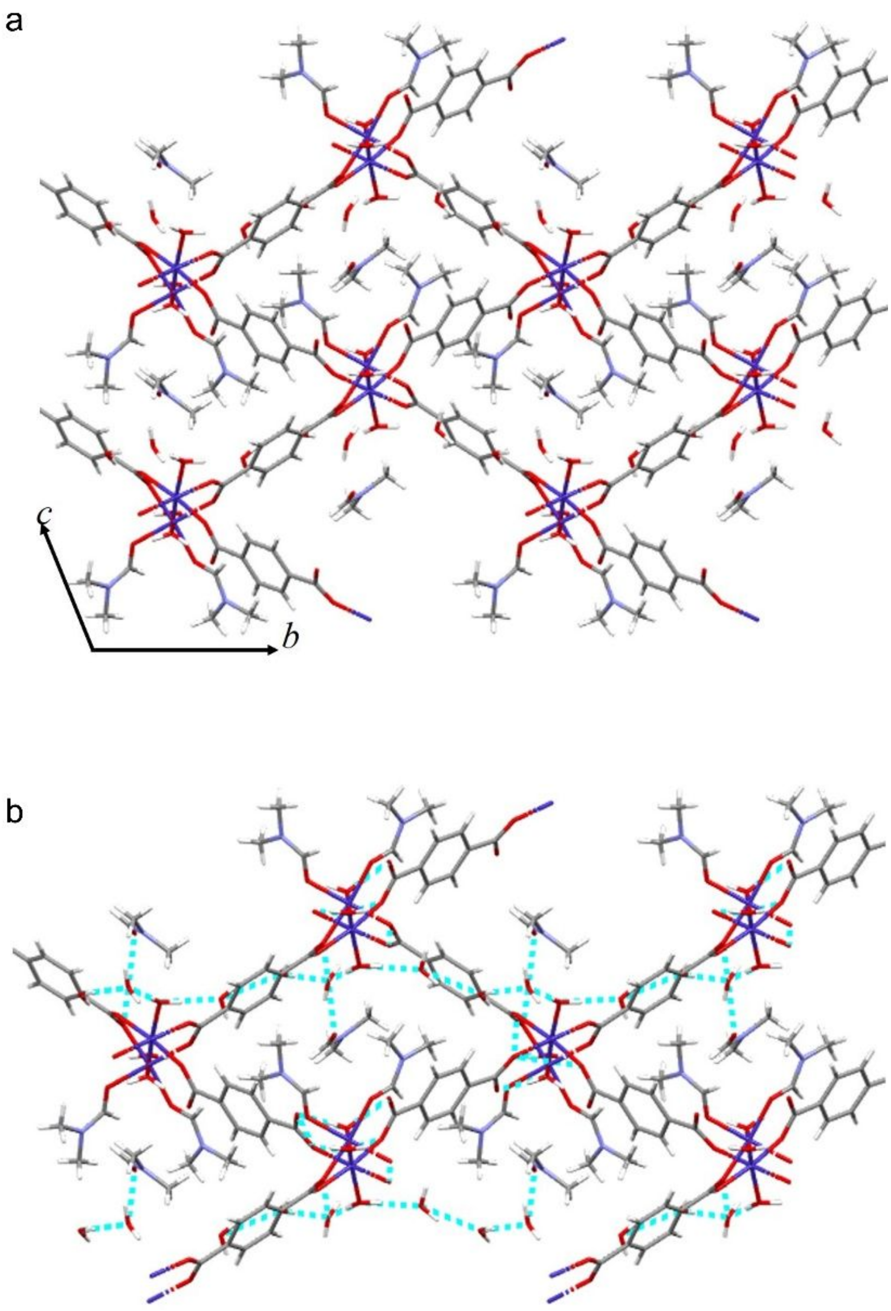

Figure S2. Molecular packing in the crystal lattice of HUST-38. C (grey), H (white), N (light blue), O (red) and Co (dark blue). Dotted lines indicate the hydrogen bonding interactions.

Supporting note 1: The crystallographic data for HUST-38 reveal that the Co1 metal centre is coordinated to two shared carboxylate ligands, each bearing a -1/2 charge, the oxidation state of Co1 is assigned as +1. Co2 metal centre is coordinated to two carboxylate ligands (each -1) and two shared carboxylate ligands (each -1/2), resulting in a total charge of -3, the oxidation state of Co2 is determined to be +3. Based on these assignments, the overall oxidation state of the cobalt centers is Co^{2+}.

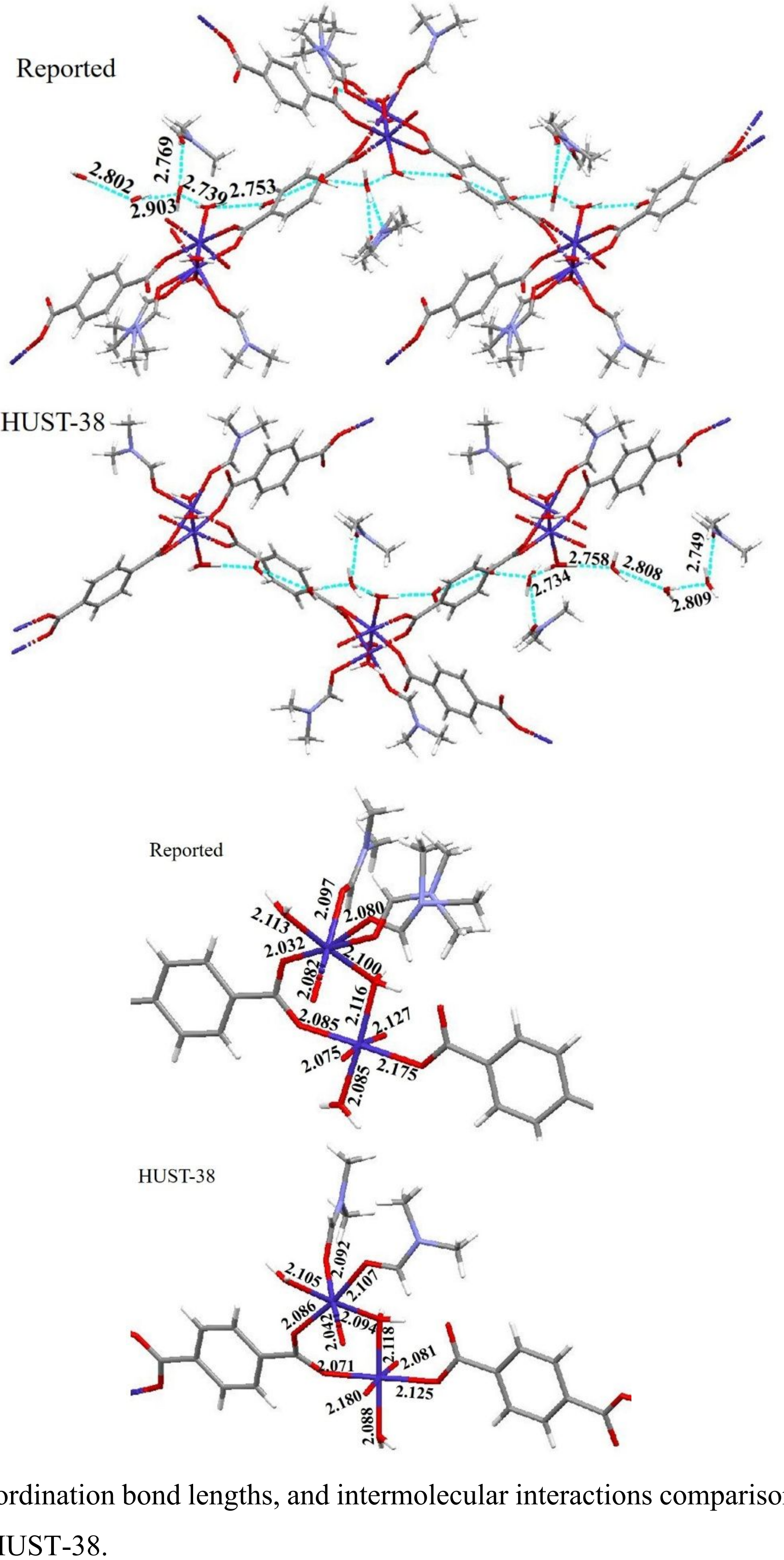

Figure S3. Coordination bond lengths, and intermolecular interactions comparison of reported structure and HUST-38.

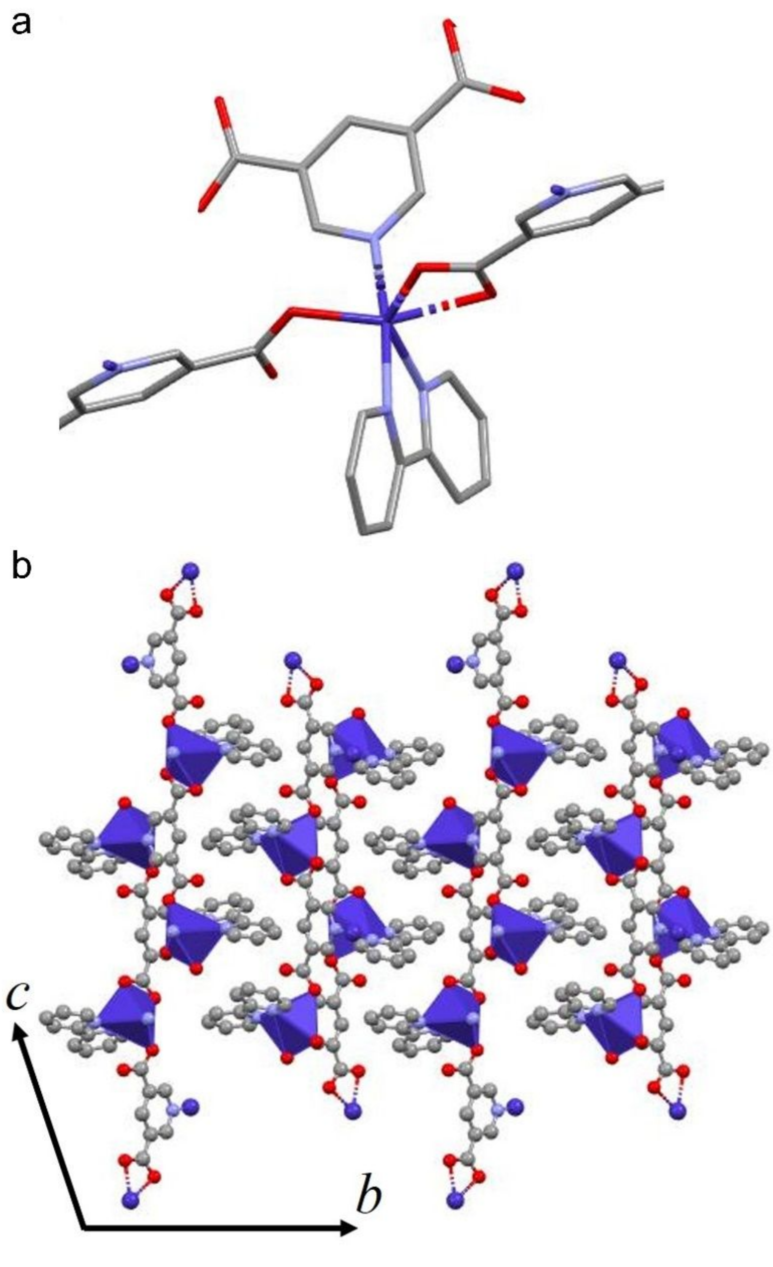

Figure S4. (a) Asymmetric unit of HUST-39. **(b)** Molecular packing in the crystal lattice of HUST-39. C (grey), H (white), N (light blue), O (red) and Co (dark blue).

Supporting note 2: For HUST-39, the Co metal centre is coordinated with two carboxylate ligands (-2), confirming that the oxidation state of Co is indeed +2.

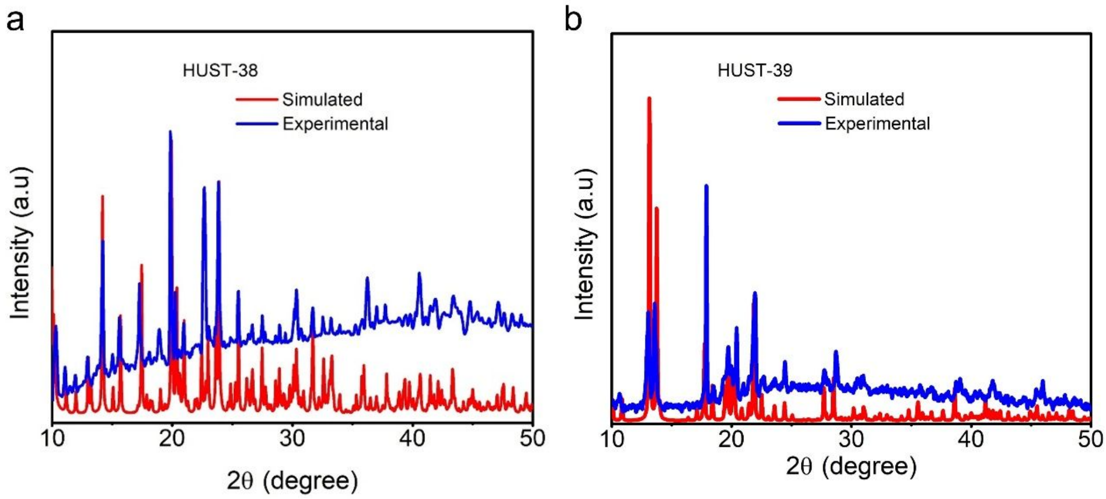

Figure S5. Experimental and simulated PXRD pattern of **(a)** HUST-38 and **(b)** HUST-39 catalyst.

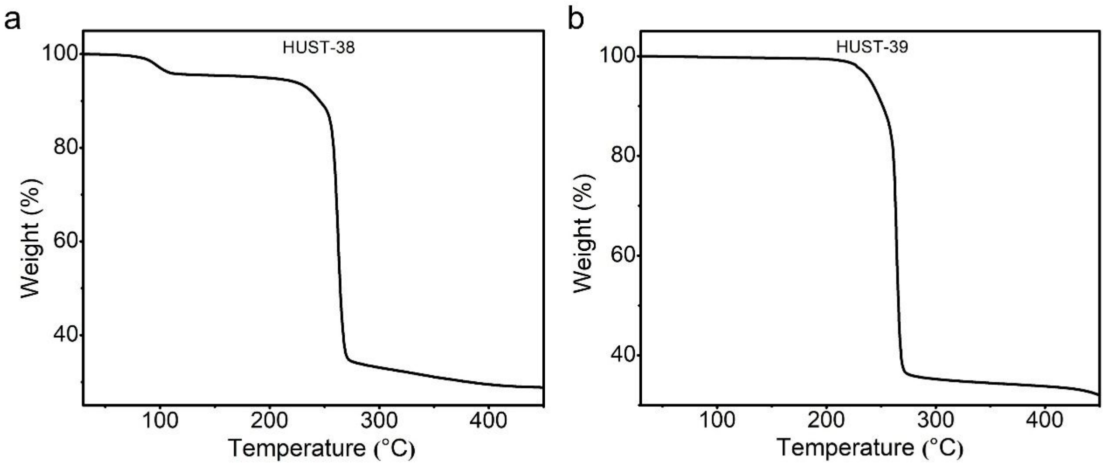

Figure S6. Thermogravimetric analysis of **(a)** HUST-38 and **(b)** HUST-39.

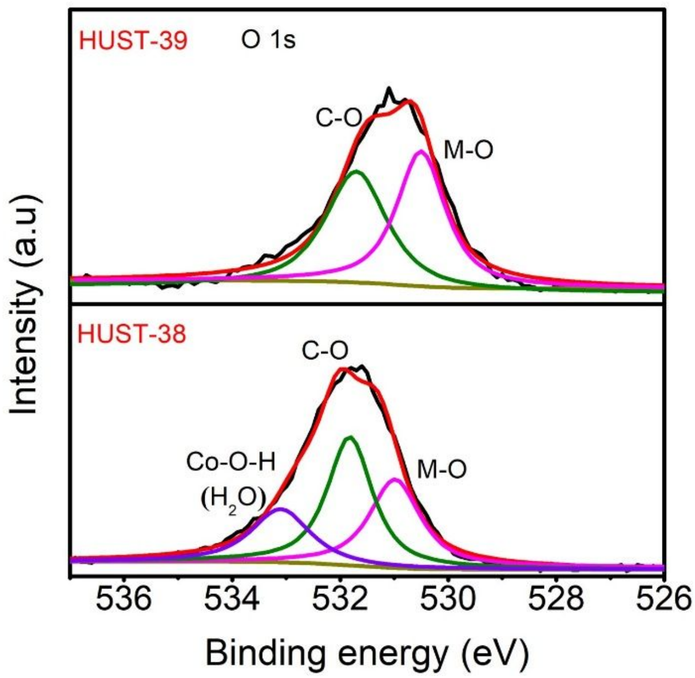

Figure S7. High resolution O1s XPS spectra of HUST-38 and HUST-39.

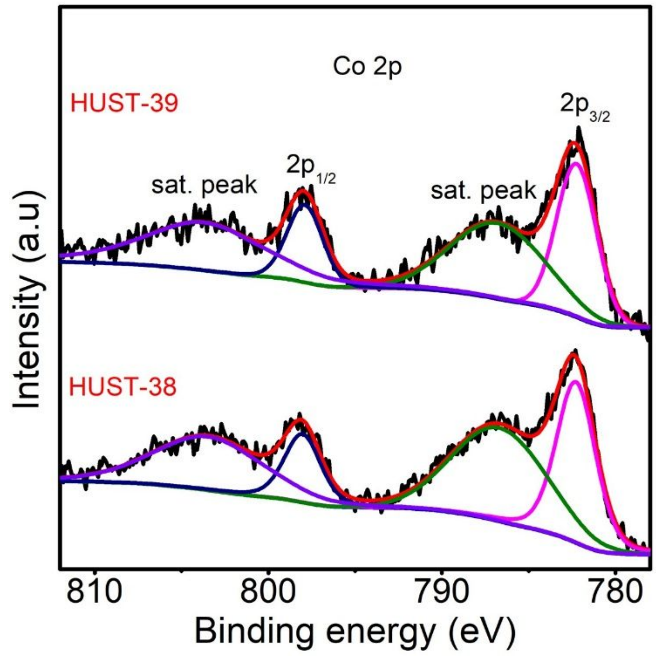

Figure S8. High resolution Co2p XPS spectra of HUST-38 and HUST-39.

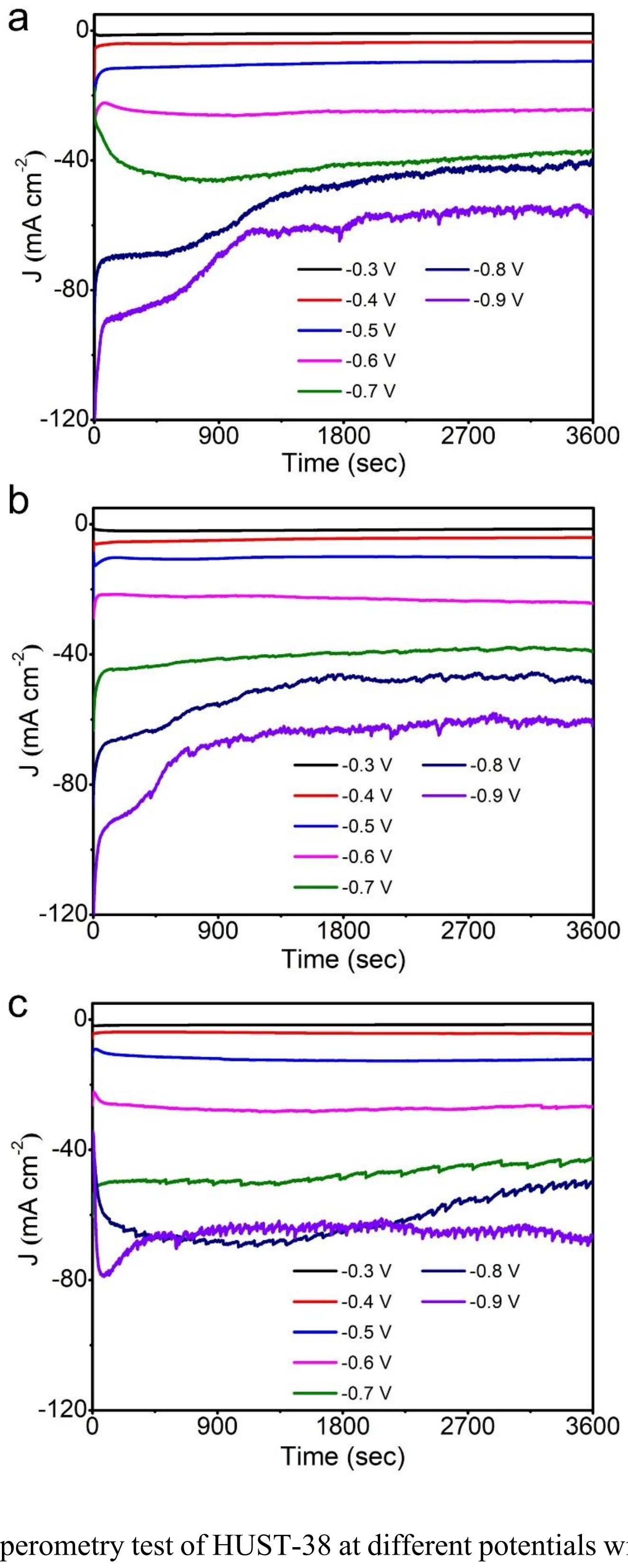

Figure S9. Chronoamperometry test of HUST-38 at different potentials with three consecutive experiments.

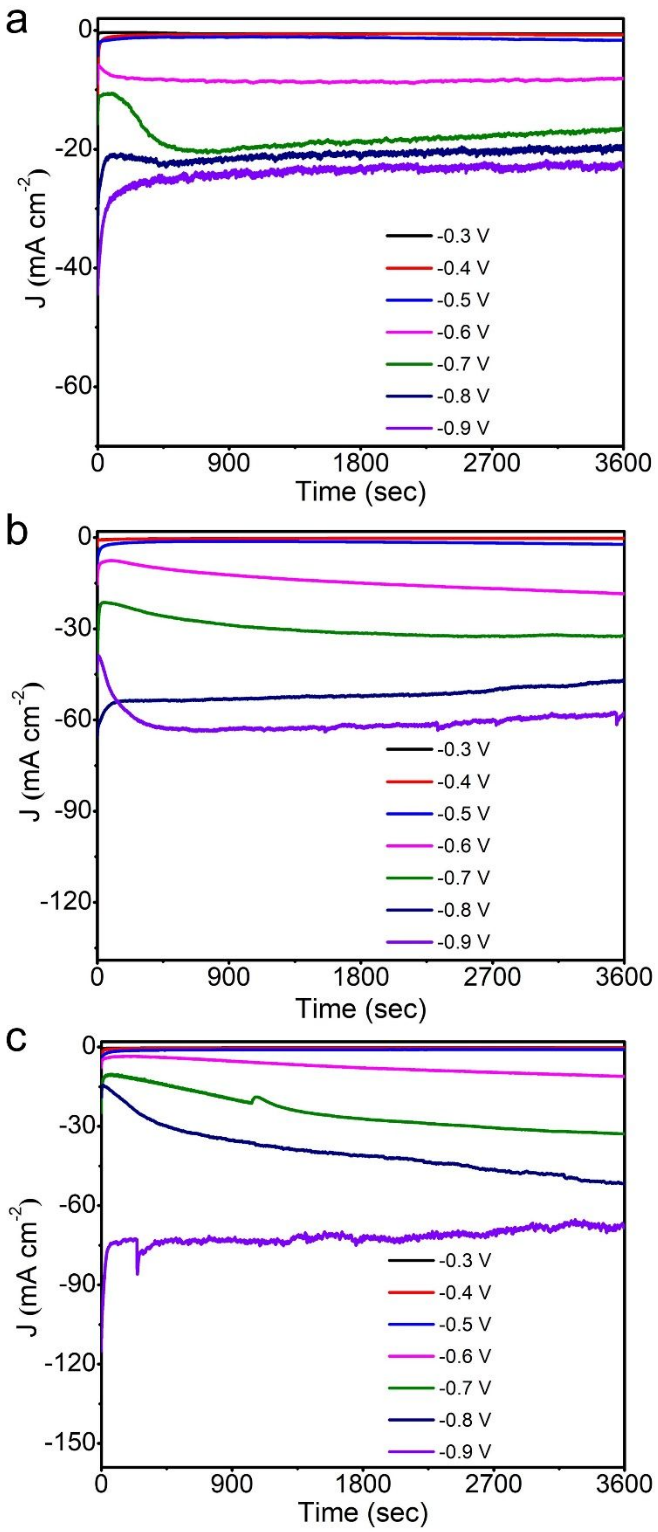

Figure S10. Chronoamperometry test of HUST-39 at different potentials with three consecutive experiments.

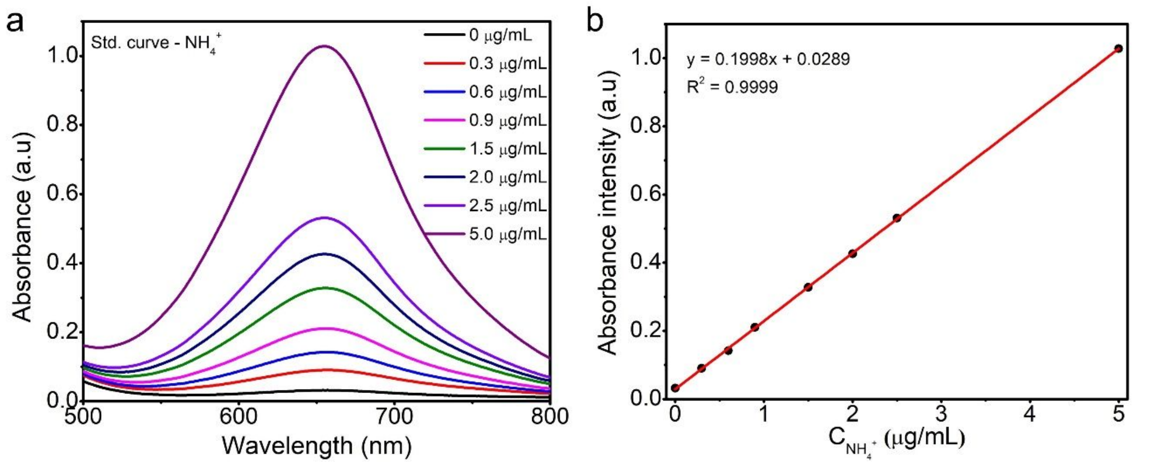

Figure S11. Ammonia detection using indophenol blue method in 0.5 M K_2SO_4. **(a)** UV-Vis. spectra of solutions with different concentration of ammonia. **(b)** The linear standard curve for the calculation of ammonia production.

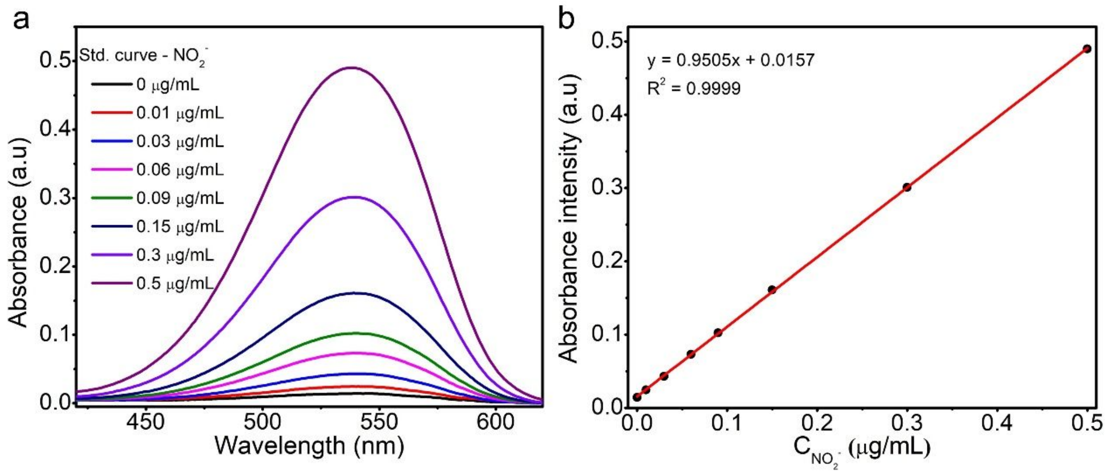

Figure S12. Nitrite detection in 0.5 M K_2SO_4. **(a)** UV-Vis. spectra of solutions with different concentration of nitrite. **(b)** The linear standard curve for the calculation of nitrite formation.

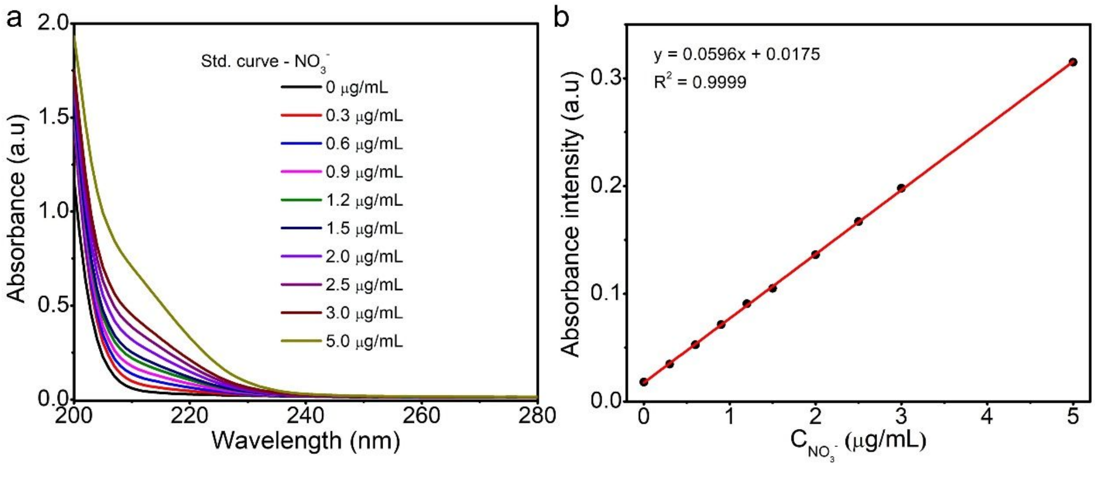

Figure S13. Nitrate detection in 0.5 M K_2SO_4. **(a)** UV-Vis. spectra of solutions with different concentration of nitrate. **(b)** The linear standard curve for the calculation of nitrate.

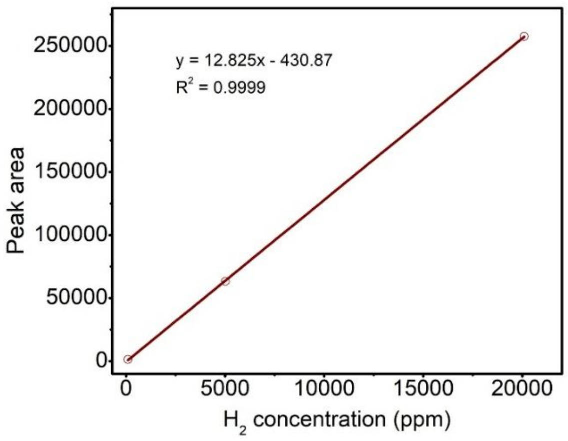

Figure S14. The linear standard curve for the calculation of H_2.

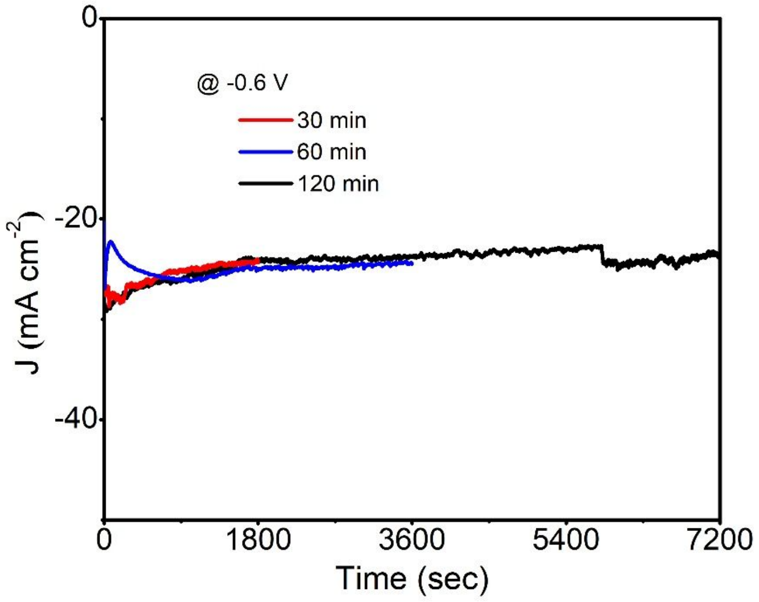

Figure S15. Chronoamperometry test of HUST-38 at -0.6 V with varying time.

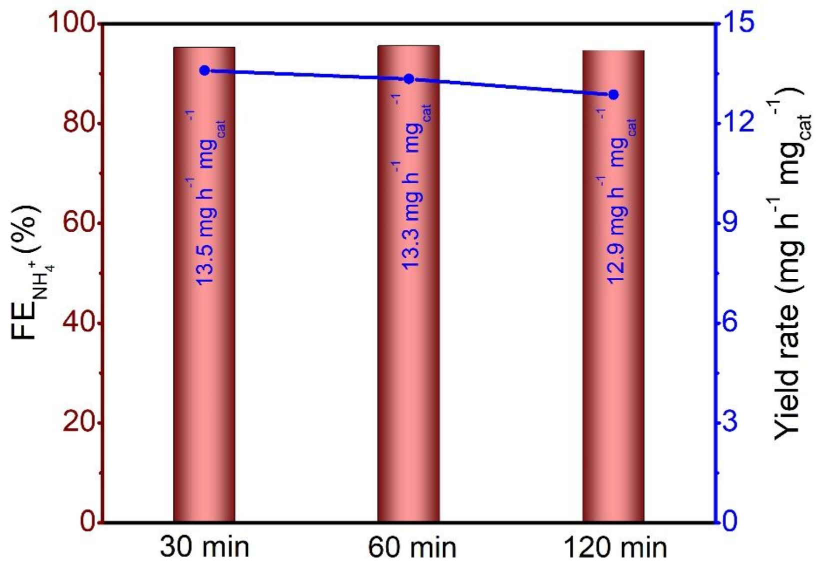

Figure S16. Faradaic efficiency and ammonia yield rate of HUST-38 at -0.6 V with varying time.

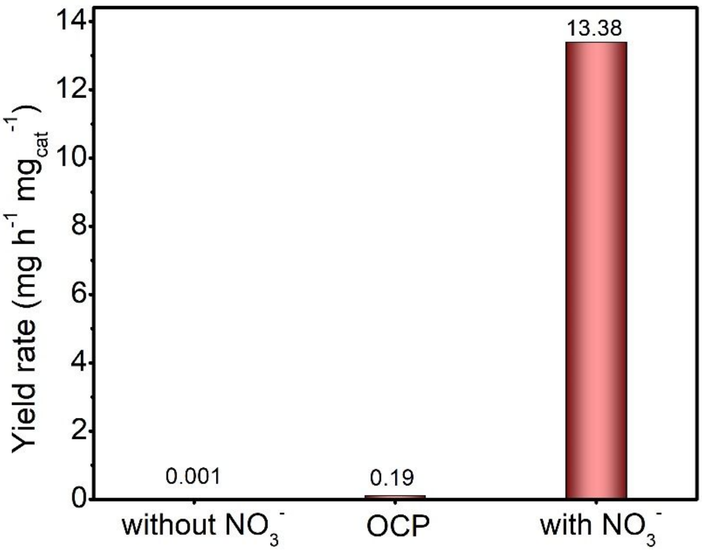

Figure S17. Control studies of HUST-38 at -0.6 V with nitrate, at open-circuit potential with nitrate and at -0.6 V without nitrate.

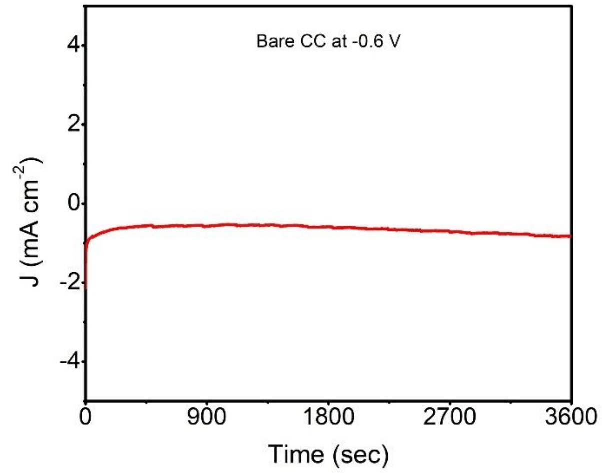

Figure S18. Chronoamperometry test of bare CC at -0.6 V with 0.5 M K_2SO_4/0.1 M NO_3^-; Obtained low yield rate of 0.03 mg h^{-1} cm^{-2}.

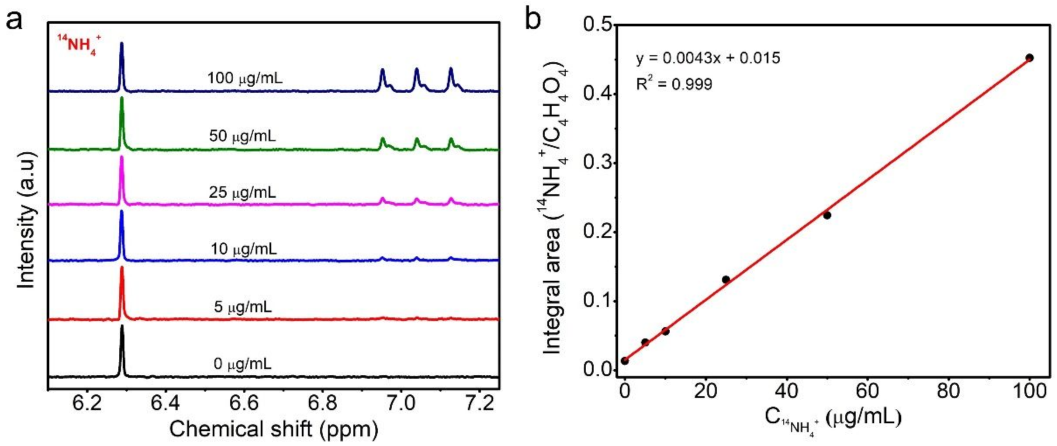

Figure S19. (a) ^{1}H-NMR spectra of $^{14}NH_4Cl$ standard solution at increasing concentrations in the range of 0-100 μg/mL. **(b)** Corresponding calibration curve obtained from the integration of the NMR signals.

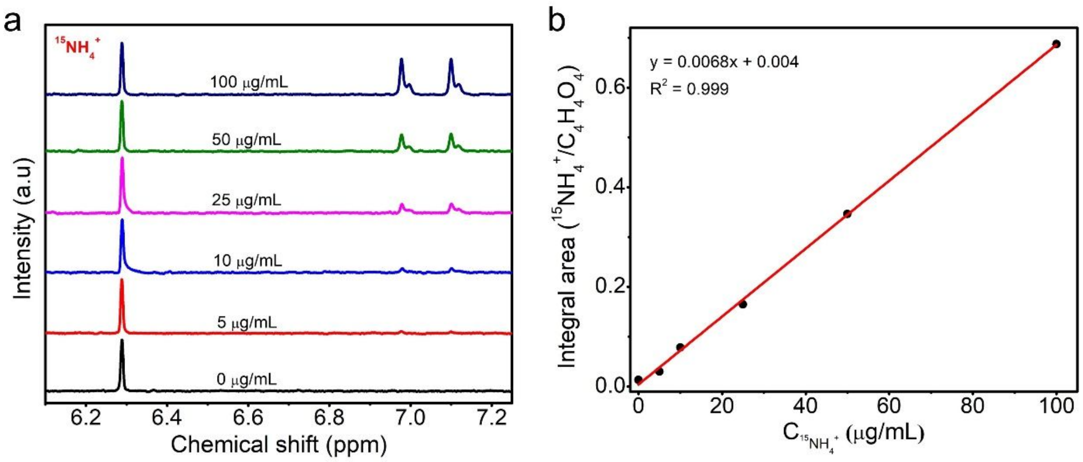

Figure S20. ^1H-NMR spectra of $^{15}NH_4Cl$ standard solution at increasing concentrations in the range of 0-100 μg/mL. **b**, Corresponding calibration curve obtained from the integration of the NMR signals.

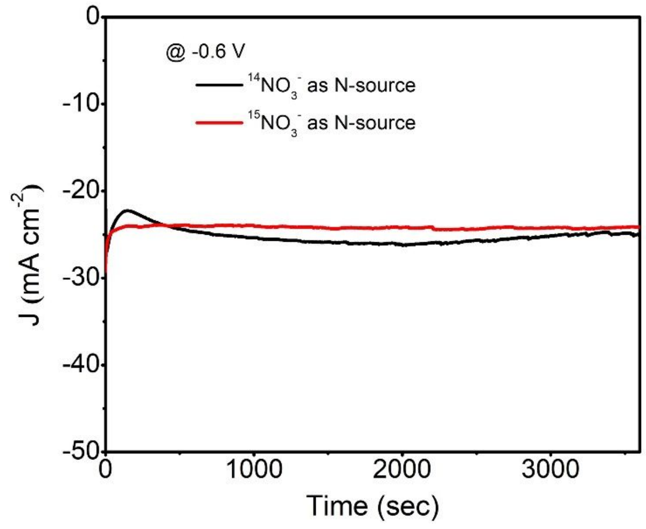

Figure S21. Chronoamperometry test of HUST-38 at -0.6 V with $^{14}NO_3^-$ and $^{15}NO_3^-$ as a nitrogen source.

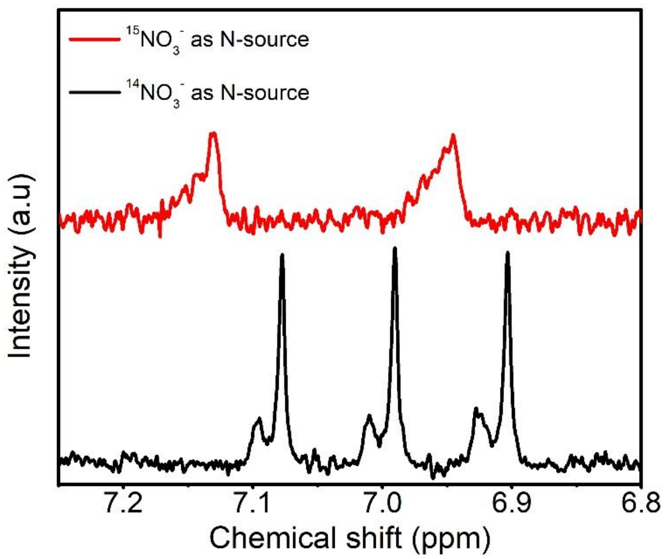

Figure S22. ^1H NMR measurements of the electrolyte with $^{14}NO_3^-$ and $^{15}NO_3^-$ as the N sources over HUST-38 at -0.6 V.

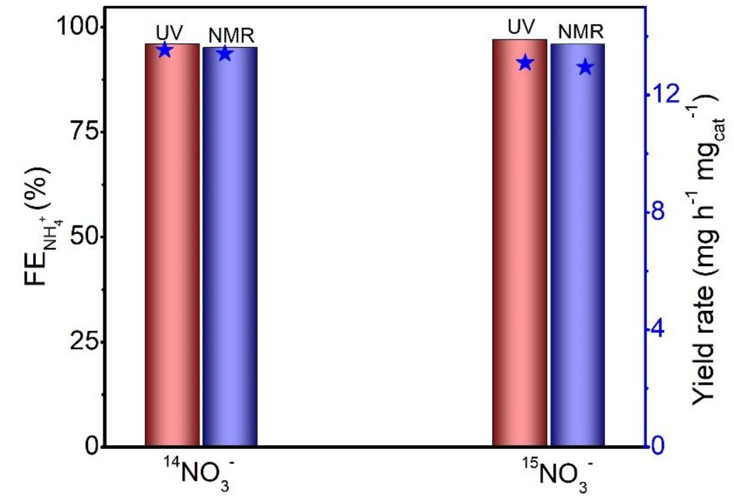

Figure S23. FE and yield rate calculation by using ^1H NMR measurements of the electrolyte with $^{14}NO_3^-$ and $^{15}NO_3^-$ as the N sources over HUST-38 at -0.6 V.

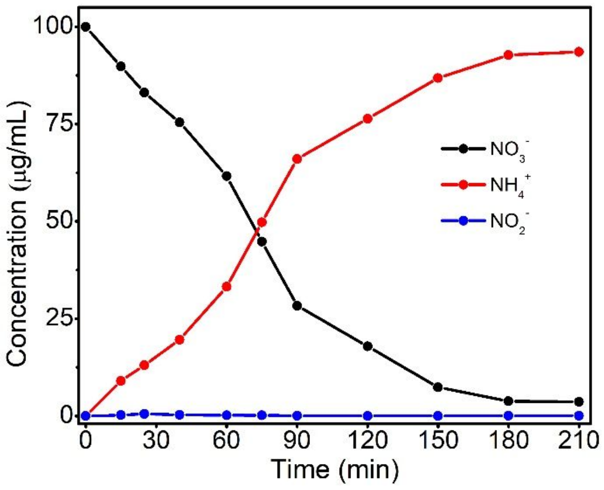

Figure S24. Time-dependent concentration variation of NO_3^-, NO_2^- and NH_4^+ during the NO_3RR electrolysis on HUST-38 at -0.6 V.

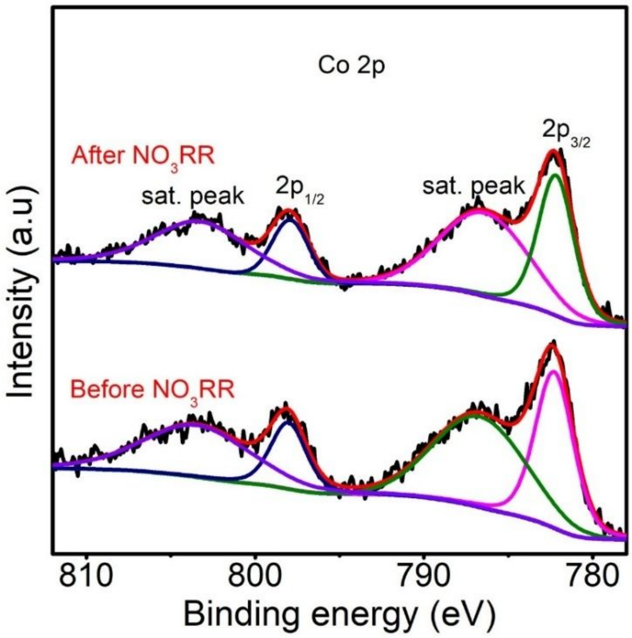

Figure S25. High resolution Co2p XPS spectra of HUST-38. Before (bottom) and after (top) NO_3RR at -0.6 V.

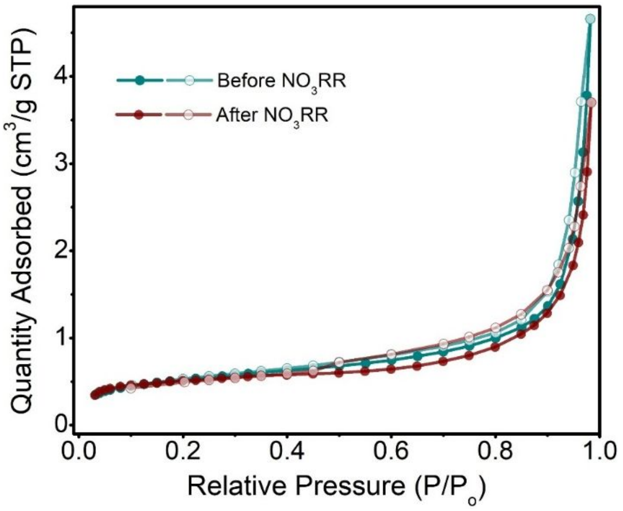

Figure S26. N_2 adsorption-desorption isotherm measurements for HUST-38 before and after NO_3RR measurements.

Supporting note 3: The electrochemical measurements were performed using a three-electrode setup, consisting of a glassy carbon electrode (GCE) loaded with the catalyst as the working electrode, a platinum foil as the counter electrode, and an Ag/AgCl electrode as the reference electrode. Chronoamperometry tests were conducted at an applied potential of -0.6 V with multiple batches. Following the electrochemical testing, the catalysts were carefully removed from the GCE, washed, and dried under vacuum conditions.

N_2 adsorption-desorption isotherm measurements revealed before and after NO_3RR showed a little bit decrease in surface area, likely due to the use of Nafion binder in preparing the catalyst ink. The Nafion binder may partially block/occupy some of the surface area, leading to a decrease in the measured BET surface area.

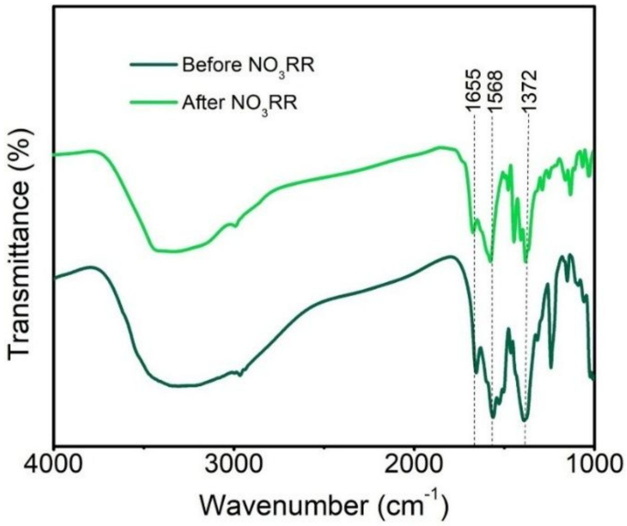

Figure S27. FT-IR spectra of HUST-38: Before (bottom) and after (top) NO_3RR at -0.6 V.

Supporting note 4: The FT-IR spectra revealed that, after catalysis, the intense peaks at 1374 and 1568 cm^{-1}, corresponding to the asymmetric and symmetric stretching vibrations of the COO^- group, were retained. The broad peak at 3400 cm^{-1} was attributed to the O-H stretching vibrations of coordinated water molecules. Additionally, a weak absorption band at 1655 cm^{-1} was observed, corresponding to the C=O stretching vibration of DMF and at 2960 cm^{-1} related to C-H stretching vibration.

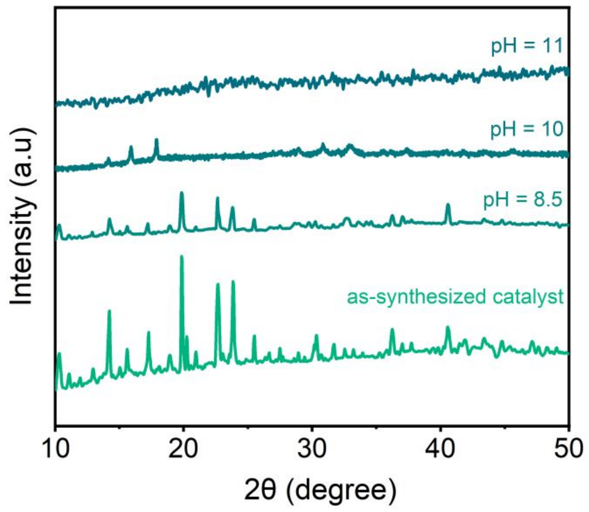

Figure S28. PXRD analysis of HUST-38 at different pH conditions.

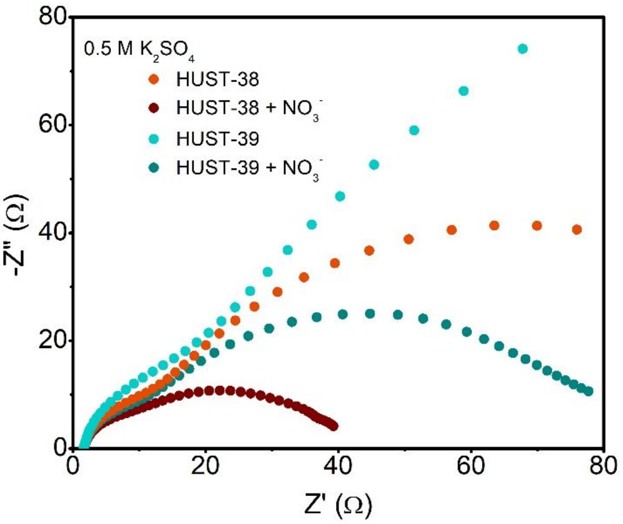

Figure S29. Electrochemical impedance spectra of HUST-38 and HUST-39 at different reaction conditions.

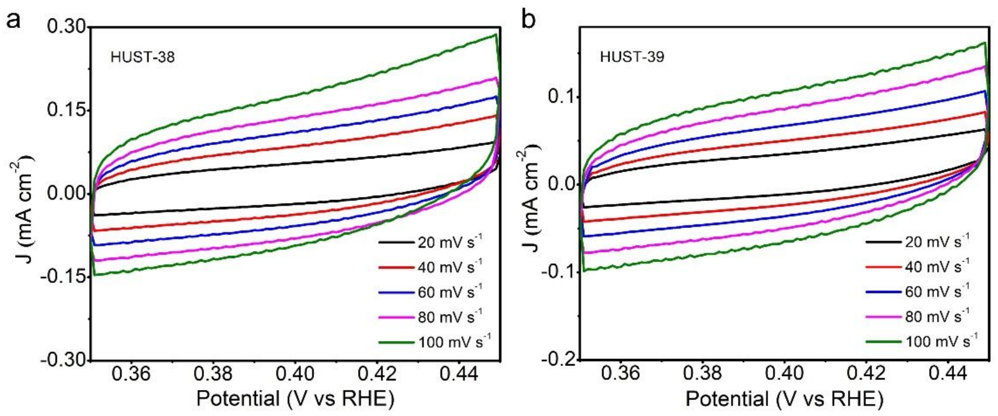

Figure S30. CV curves of **(a)** HUST-38 and **(b)** HUST-39 collected at different scan rates.

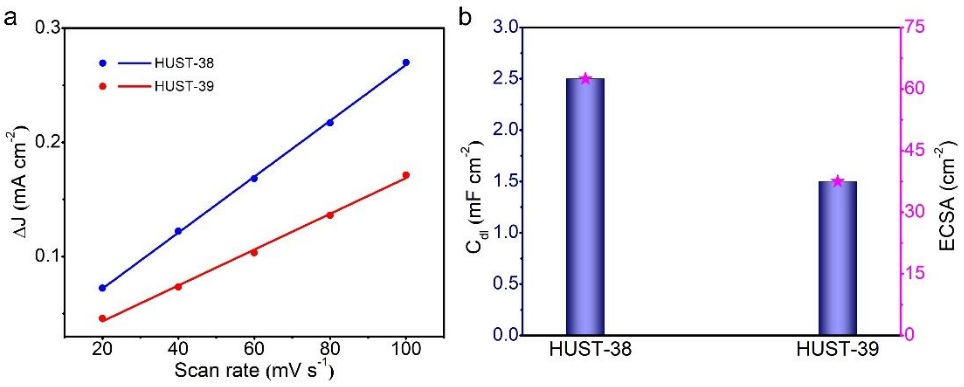

Figure S31. (a) Double layer capacitances (C_{dl}) and **(b)** electrochemical surface area (ECSA) of HUST-38 and HUST-39.

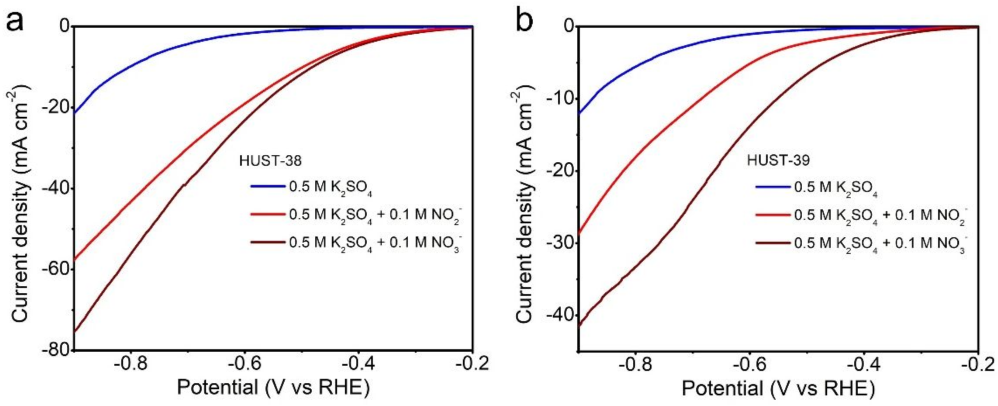

Figure S32. LSV curves of different reaction medium at -0.6 V: **(a)** HUST-38. **(b)** HUST-39.

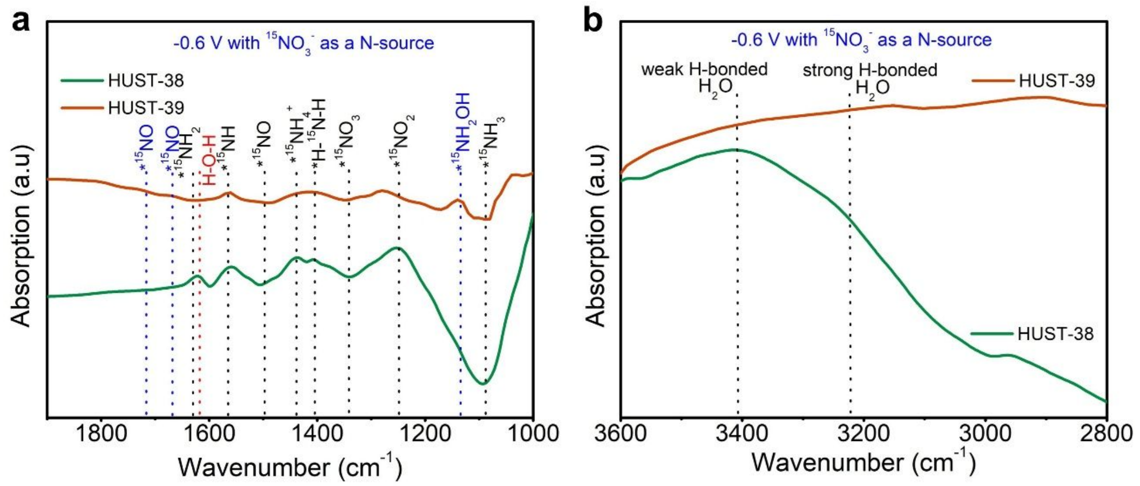

Figure S33. In situ FT-IR spectra **(a, b)** of HUST-38 and HUST-39 in 0.5 M K_2SO_4/0.1 M $^{15}NO_3^-$ at -0.6 V.

Supporting note 5: As a result of the isotope effect (^{15}N labelling), all N-containing intermediates exhibited shifts to lower wavenumbers (17-30 cm^{-1}), forming $*^{15}NH_4^+$.[3] Specifically, the peak at 1339 cm^{-1} is attributed to the stretching vibration of $*^{15}NO_3^-$ ions, while the positive peak at 1438 cm^{-1} confirms the formation of $*^{15}NH_4^+$, indicating successive deoxygenation and hydrogenation of NO_3^-. Furthermore, the overlap $*H_2O$ (H-O-H) peaks at 1620 cm^{-1} indicates the formation of active hydrogen intermediates and the vibrational band at 3350-3450 cm^{-1} attributed for the weakly H-bonded H_2O molecules for HUST-38, whereas these two peaks were not formed in HUST-39. Similarly, the band at 1135 cm^{-1}, associated with N-H stretching vibration of hydroxylamine ($*^{15}NH_2OH$) intermediates, appears only in HUST-39, further supporting our claims in this study. These experiments provided additional evidence for the proposed reaction pathway.

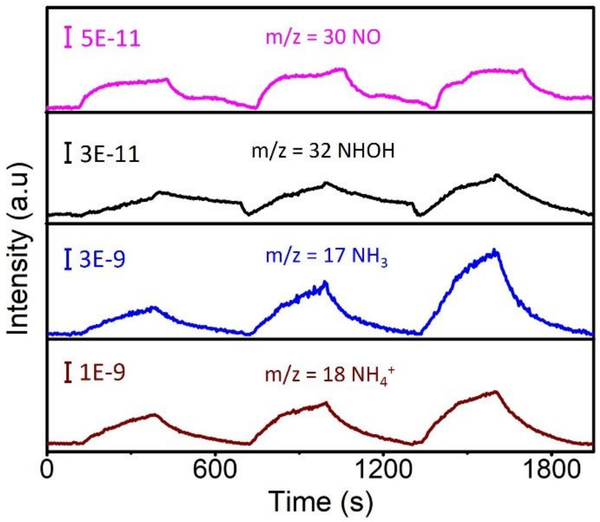

Figure S34. Product analysis. In situ DEMS measurement of HUST-38 in 0.5 M K_2SO_4/0.1 M NO_3^- at -0.6 V.

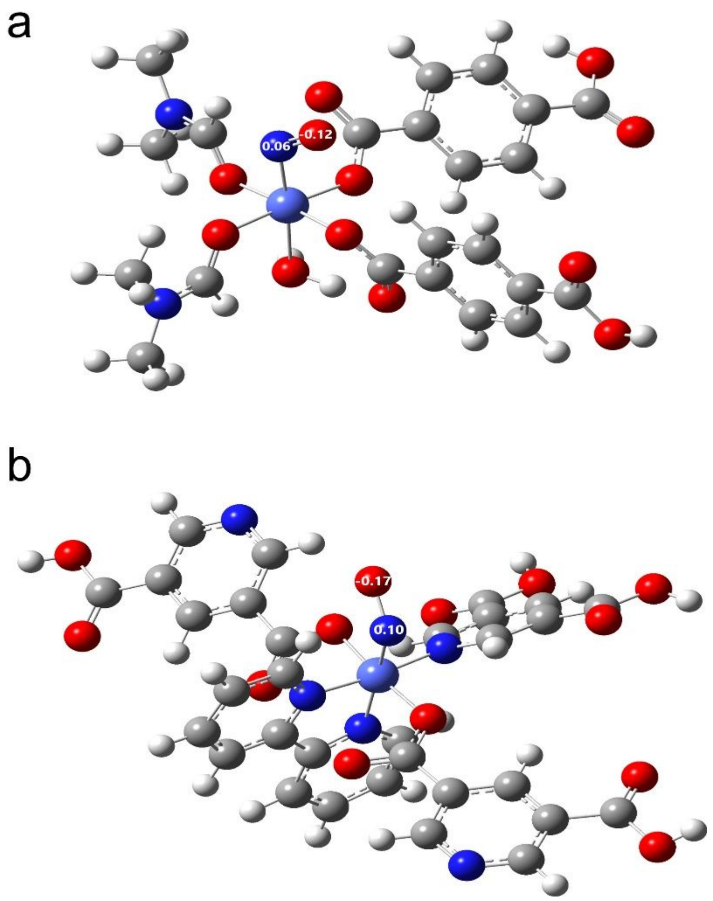

Figure S35. Electron charge analysis for **(a)** HUST-38 and **(b)** HUST-39 for the formation of *NO intermediate

Supporting note 6: The incoming moiety in both cases (either NHO or NOH pathway) is a proton (H^+). Notably, the nitrogen atom bonded to Co and O atom stabilized by its surrounding environment for HUST-38. The Co center, coordinated to four oxygen atoms, engages in back-donation to nitrogen, reducing its positive charge and facilitating its availability for protonation.

In contrast, HUST-39 exhibits a distinct coordination sphere, where three nitrogen and two oxygen atoms are bonded to Co. Here, the π-electrons or non-bonding electrons from the NO nitrogen are directed towards the empty d-orbitals of Co, while the oxygen atom of NO withdraws electrons from nitrogen. Consequently, the nitrogen atom in Co-NO bears a high positive charge, diminishing its electron-donating capacity for incoming proton (H+) and hindering N-H-O bond formation.

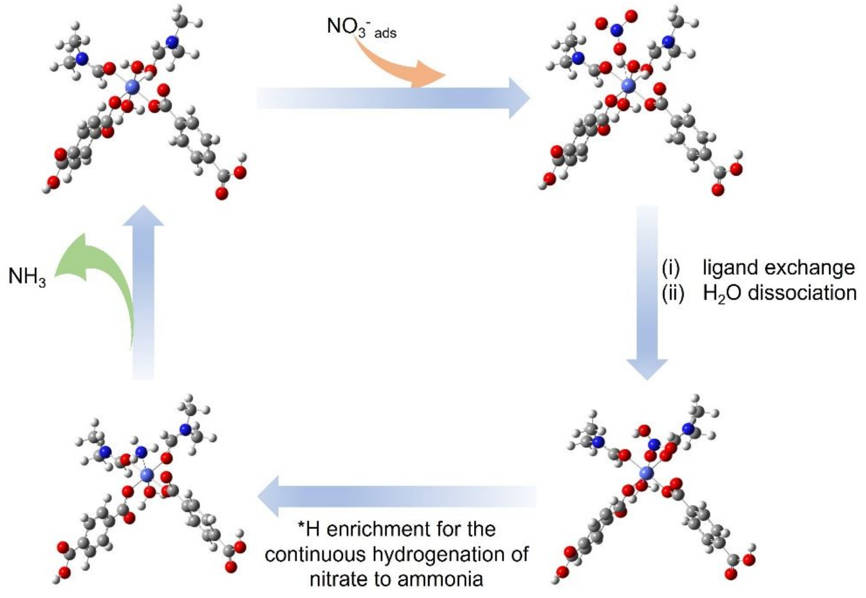

Figure S36. Schematic representation of the possible route to convert NO_3^- into NH_3 over HUST-38 catalyst.

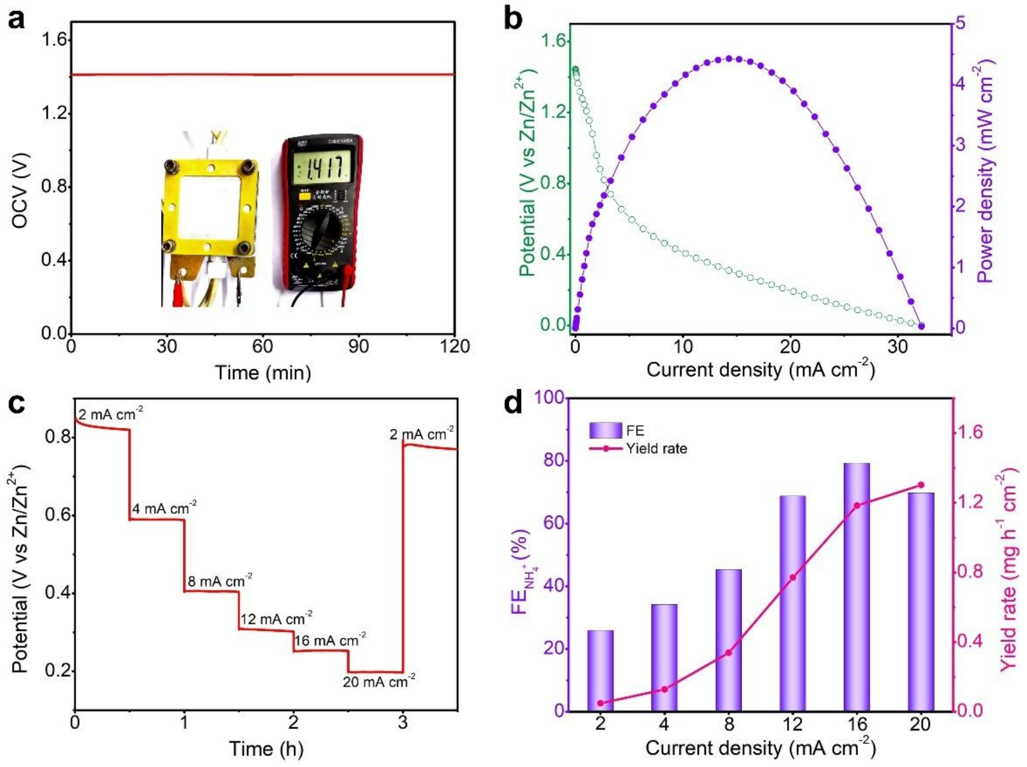

Figure S37. (a) Open-circuit voltage curve. **(b)** Discharging polarization curve and power density of HUST-38. **(c)** Discharging measurements at different current densities. **(d)** NH_3 FEs and yields of the Zn-NO_3^- battery at different current densities using HUST-38 as cathode**.**

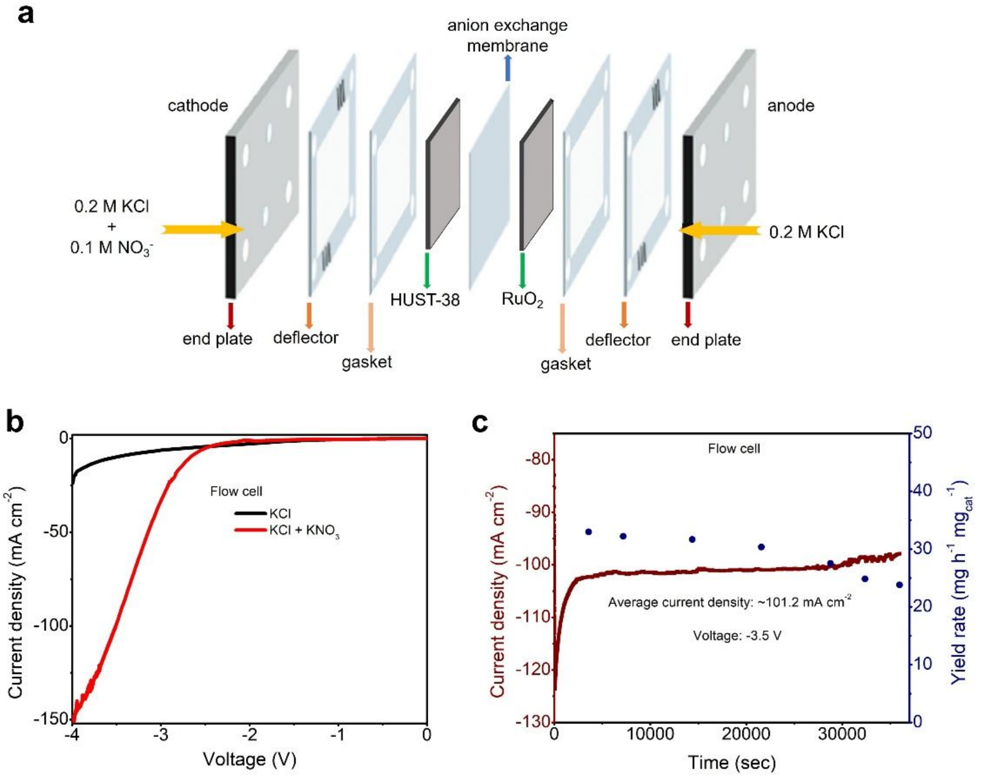

Figure S38. (a) Schematic illustration of the flow cell electrolyser. **(b)** Polarization curves and **(c)** Chronoamperometric curves of HUST-38 catalyst for nitrate to ammonia conversion.

Supporting Note 7: Electrolytic cell architecture was fabricated with HUST-38 as cathode and RuO_2 as the anode with 0.2 M KCl/0.1 M NO_3^- and 0.2 M KCl serving as the catholyte (500 mL) and anolyte (500 mL) respectively with anion exchange membrane (Figure S38a). Polarization curves revealed HUST-38 catalyst reached a current of 150 mA cm^{-2} at a cell voltage of -4 V (Figure S38b). HUST-38 electrocatalyst effectively converts the nitrate into ammonia at the average current density of 101.2 mA cm^{-2} at the applied cell voltage of -3.5 V, with complimentary counter reaction (OER) at the anodic chamber (Figure S38c).

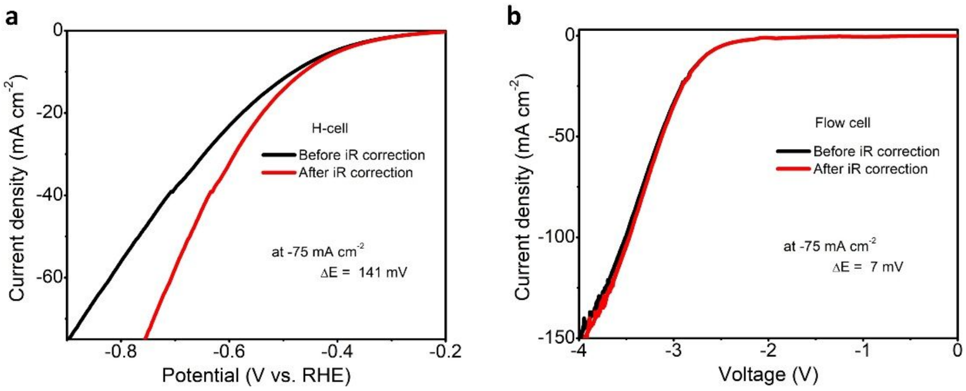

Figure S39. iR comparison of HUST 38 in **(a)** H-cell and **(b)** Flow-cell measurements.

Table S1: Unit cell parameter comparison for the reported structure and HUST-38.

Cell parameter (Å)	Reported structure	HUST-38
a	9.05420(19)	9.0070(18)
b	17.3492(4)	17.492(4)
c	10.6114(2)	10.486(2)

Table S2: Faradaic efficiency for HUST-38 catalyst across 3 independent batches.

Potential (V vs RHE)	FE (%) 1	FE (%) 2	FE (%) 3
-0.3	84.00	85.94	82.91
-0.4	88.58	89.34	87.70
-0.5	91.30	93.13	91.14
-0.6	95.52	96.26	95.33
-0.7	92.85	91.08	88.76
-0.8	91.20	89.88	86.69
-0.9	87.90	84.98	84.94

Table S3: Faradaic efficiency for HUST-39 catalyst across 3 independent batches.

Potential (V vs RHE)	FE (%) 1	FE (%) 2	FE (%) 3
-0.3	40.10	45.08	47.23
-0.4	59.20	49.69	56.01
-0.5	63.73	58.23	65.75
-0.6	72.39	69.75	69.95
-0.7	59.19	60.19	59.40
-0.8	55.17	56.65	54.14
-0.9	50.64	51.43	46.17

Table S4: Electrocatalytic NO_3RR Performance of HUST-38 with detectable products under various applied potential.

Applied Potential (V)	Detected Products	Quantification Method	FE
-0.3	NH_4^+	Indophenol Blue	84.28278
	NO_2^-	Griess Assay	10.14923
	H_2	GC-MS	4.84416
-0.4	NH_4^+	Indophenol Blue	88.54046
	NO_2^-	Griess Assay	7.36918
	H_2	GC-MS	3.63267
-0.5	NH_4^+	Indophenol Blue	91.85438
	NO_2^-	Griess Assay	1.63588
	H_2	GC-MS	3.53121
-0.6	NH_4^+	Indophenol Blue	95.70457
	NO_2^-	Griess Assay	0.43231
	H_2	GC-MS	2.89555
-0.7	NH_4^+	Indophenol Blue	90.89609
	NO_2^-	Griess Assay	0.43167
	H_2	GC-MS	7.55308
-0.8	NH_4^+	Indophenol Blue	89.25779
	NO_2^-	Griess Assay	0.37618
	H_2	GC-MS	9.1826
-0.9	NH_4^+	Indophenol Blue	85.94652
	NO_2^-	Griess Assay	0.20657
	H_2	GC-MS	12.77947

Table S5: Comparison of the electrocatalytic NO_3RR performance of HUST-38 with recently reported electrocatalysts.

Catalyst	Electrolyte	FE (%)	YR (mg h^{-1} mg_{cat}^{-1})	Potential (V vs RHE)	Reference
**HUST-38**	0.5 M K_2SO_4 + 0.1 M NO_3^-	**95.7**	**13.33**	**-0.6**	***This work***
Co_3O_4@CoBi/CC	0.1 M PBS + 0.1 M $NaNO_3$	97	1.97	-0.7	*6*
$Co_{1\text{-}P}$/NPG	0.5 M K_2SO_4 + 0.1 M KNO_3	93.8	8.6	-0.7	*7*
Fe SAC	0.1 M K_2SO_4 + 0.5 M KNO_3	75	0.55	-0.66	*8*
CoNi@NC	1 M KOH + 0.1 M KNO_3	93	2.856	-0.1	*9*
Fe/Cu	1 M KOH + 0.1 M KNO_3	92.51	18.36	-0.5	*10*
Cu_7Ni_3/OMC	0.1 M PBS + 500 ppm KNO_3	78.9	0.2374	-0.4	*11*
Fe-Co_3O_4 NA/TM	0.1 M PBS + 50 mM KNO_3	95.5	0.624	-0.7	*12*
$Fe_{0.36}Cu_{0.64}$	0.1 M Na_2SO_4 15 mM KNO_3	92	14.08	-0.6	*13*
O-Cu–PTCDA	0.1 M PBS + 500 ppm of NO_3^-	77	2.213	-0.4	*14*
PA-RhCu cNCs	0.1 M $HClO_4$ + 0.05 mM KNO_3	93.7	2.4	+0.05	*15*
CuPc@MXene	0.5 M Na_2SO_4 + 30 mg L^{-1} NO_3^-–N	94	2.72	-1.06	*16*

Table S6: Stability comparison of the *e*-NO_3RR performance of HUST-38 with recently reported MOFs and molecular materials-based catalysts.

**Catalyst**	**Potential (V vs RHE)**	**FE (%)**	**Stability cycle (n)**	**Total stability time (h)**	**Reference**
**HUST-38**	**-0.6**	95.7	**32**	**96**	***This work***
Cu-FA	-0.9	98.17	10	~20	*17*
NiFe-MOF	-0.6	90.8	20	20	*18*
Co-MOF/$NiMo_6$	-0.8	98.2	6	12	*19*
PCC-Co	-0.85	~85	10	10	*20*
NJUZ-2 ([Co_2(TCPPDA)(H_2O)$_5$]·(H_2O)$_9$(DMF))	-0.8	~91	7	70	*21*
Ni-MOF-Ru	-0.6	91.5	20	20	*22*
Cu–N_4B_2	-0.6	98.2	6	30	*23*
Fe(TCNQ)$_2$	-1.1	85.2	10	10	*24*
CuTABQ	-0.4	97.7	10	10	*25*
NiO_4-CCP	-0.7	94.7	8	8	*26*
Fe_2Co-MOF	-1.1	90.55	10	10	*27*
Cu_1Co_1HHTP	-0.6	96.4	7	3.5	*28*
PCN-250-Fe_3 MOF	-1.0	~90	6	6	*29*
NiCoBDC@ HsGDY	-0.34	99.1	16	16	*30*
UiO-CuZn	-1.0	91.4	10	20	*31*
FeCo PBA HCAs	-0.6	81.01	5	30	*32*
ZIFNC@GDY	**-0.745**	98.51	10	10	*33*
Cu@CuHHTP	-0.95	58.13	5	10	*34*

Table S7: ICP analysis of HUST-38 before and after *e*-NO_3RR.

Sample	Sampling mass/g	Constant volume/mL	Dilution factor	Measured elements	Instrument readings (mg/L)	Conversion content (mg/kg)	Mass fraction (%)
Sample 1 (Before NO_3RR)	0.0233	25	50	Co	2.4060	129078.3	12.9078
Sample 2 (After NO_3RR)	0.0233	25	50	Co	2.3984	128667.4	12.8667

Table S8: R_s and R_{CT} values of HUST-38 and HUST-39 at different reaction conditions.

**Catalyst**	**Electrolyte**	**R_s (Ω)**	**R_{CT} (Ω)**
HUST-38	0.5 M K_2SO_4 + 0.1 M NO_3^-	1.65	38.06
HUST-39	0.5 M K_2SO_4 + 0.1 M NO_3^-	1.66	82.41
HUST-38	0.5 M K_2SO_4	1.69	141.37
HUST-39	0.5 M K_2SO_4	1.68	391.83

Table S9: Performance comparisons of HUST-38 with previously reported Zn-NO_3^- batteries.

**Catalyst**	**Power density ($mW\ cm^{-2}$)**	**YR ($mg\ h^{-1}\ cm^{-2}$)**	**Ref**
**HUST-38**	**4.42**	**1.18**	***This work***
LPNBSC (($La_{0.2}Pr_{0.2}Nd_{0.2}Ba_{0.2}Sr_{0.2}$)$CoO_{3-\delta}$)	9.3	0.96	*35*
Ni-MOF-Ru	4.99	2.1	*36*
FeMoN@C NO	3.23	0.07	*37*
Cu/Cu_2O/Pi NWs	3.89	0.69	*38*
Co-B@CoOx	4.78	0.89	*39*
CoNi-Vp-1.0	1.05	0.21	*40*
NiCoBDC@HsGDY	3.66	1.12	*41*
Ni-NPs-1.6	4.2	NA	*42*
0.6W-O-CoP	9.27	2.79	*43*
$NiCo_2O_4$/CC	3.94	0.82	*44*
v_{Co}-Co_3O_4/CC	8.1	1.86	*45*
Co_2B@Co_3O_4/TM	3.21	0.74	*46*
Fe/Ni_2P	3.25	0.38	*47*

References

(1) Chen, G.-F.; Yuan, Y.; Jiang, H.; Ren, S.-Y.; Ding, L.-X.; Ma, L.; Wu, T.; Lu, J.; Wang, H. Electrochemical reduction of nitrate to ammonia via direct eight-electron transfer using a copper–molecular solid catalyst. *Nat. Energy* **2020**, *5*, 605-613.

(2) Wang, Y.; Zhou, W.; Jia, R.; Yu, Y.; Zhang, B. Unveiling the Activity Origin of a Copper based Electrocatalyst for Selective Nitrate Reduction to Ammonia. *Angew. Chem. Int. Ed.* **2020**, *59*, 5350-5354.

(3) Han, S.; Li, H.; Li, T.; Chen, F.; Yang, R.; Yu, Y.; Zhang, B. Ultralow overpotential nitrate reduction to ammonia via a three-step relay mechanism. *Nat. Catal.* **2023**, *6*, 402-414.

(4) Kim, K.-H.; Lee, H.; Huang, X.; Choi, J. H.; Chen, C.; Kang, J. K.; O'Hare, D. Energy-efficient electrochemical ammonia production from dilute nitrate solution. *Energy Environ. Sci.* **2023**, *16*, 663-672.

(5) Smidstrup, S.; Markussen, T.; Vancraeyveld, P.; Wellendorff, J.; Schneider, J.; Gunst, T.; Verstichel, B.; Stradi, D.; Khomyakov, P. A.; Vej-Hansen, U. G. QuantumATK: an integrated platform of electronic and atomic-scale modelling tools. *J. Phys. Condens. Matter* **2020**, *32*, 015901.

(6) Xie, L.; Liu, Q.; Li, X.; Wang, G.; Ren, Z.; Qiu, H.; Sun X.; Kong, Q. Electrochemical Reduction of Nitrate to Ammonia on an In Situ-Derived Co_3O_4@CoBi Core−Shell Nanoarray. *ACS Appl. Nano Mater.* **2024**, *7*, 951−956.

(7) Ni, J.; Yan, J.; Li, F.; Qi, H.; Xu, Q.; Su, C.; Sun, L.; Sun, H.; Ding, J.; Liu, B. Atomic Co─P Catalytic Pair Drives Efficient Electrochemical Nitrate Reduction to Ammonia. *Adv. Mater.* **2024**, 14, 2400065.

(8) Wu, ZY.; Karamad, M.; Yong, X.; Huang, Q.; Cullen, D. A.; Zhu, P.; Xia, C.; Xiao, Q.; Shakouri, M.; Chen, F.-Y.; Kim, J. T.; Xia, Y.; Heck, K.; Hu, Y.; Wong, M. S.; Li, Q.; Gates, I.; Siahrostami, S.; Wang, H. Electrochemical ammonia synthesis via nitrate reduction on Fe single atom catalyst. *Nat. Commun.* **2021**, *12*, 287.

(9) Lei, F.; Zhang, Y.; Xu, M.; Li, K.; Zhang, M.; Huai, R.; Xie, J.; Hao, P.; Cui G.; Tang, B. Energy-Efficient Ammonia Synthesis from Nitrate via CoNi Alloys Incorporated in Carbon Frameworks. *ACS Sustainable Chem. Eng.* **2023**, *11*, 9057−9064.

(10) Zhang, S.; Wu, J.; Zheng, M.; Jin, X.; Shen, Z.; Li, Z.; Wang, Y.; Wang, Q.; Wang, X.; Wei, H.; Zhang, J.; Wang, P.; Zhang, S.; Yu, L.; Dong, L.; Zhu, Q.; Zhang, H.; Lu, J. Fe/Cu diatomic catalysts for electrochemical nitrate reduction to ammonia. *Nat. Commun.* **2023**, *14*, 3634.

(11) Zhao, J.; Liu, L.; Yang, Y.; Liu, D.; Peng, X.; Liang S.; Jiang, L. Insights into Electrocatalytic Nitrate Reduction to Ammonia via Cu-Based Bimetallic Catalysts. *ACS Sustainable Chem. Eng.* **2023**, *11*, 2468−2475.

(12) Wei, P.; Liang, J.; Liu, Q.; Xie, L.; Tong, X.; Ren, Y.; Li, T.; Luo, Y.; Li, N.; Tang, B.; Asiri, A. M.; Hamdy, M. S.; Kong, Q.; Wang, Z.; Sun, X. Iron-doped cobalt oxide nanoarray for efficient electrocatalytic nitrate-to-ammonia conversion. *J. Coll. Int. Sci.* **2022**, *615*, 636−642.

(13) Shih, Y.-J.; Wu, Z.-L.; He, Y.-C. Tuning transition metals layered-electroplated on bimetallic M_xCu_{1-x} crystallites (M = Fe, Co, Ni, and Zn) to boost ammonia yield in electrocatalytic reduction of nitrate wastewaters. *J. Hazard. mater.* **2024**, *477*, 135276.

(14) Chen, GF.; Yuan, Y.; Jiang, H.; Ren, S.-Y.; Ding, L.-X.; Ma, L.; Wu, T.; Lu, J.; Wang, H. Electrochemical reduction of nitrate to ammonia via direct eight-electron transfer using a copper–molecular solid catalyst. *Nat. Energy* **2020**, *5*, 605–613.

(15) Ge, Z.-X.; Wang, T.-J.; Ding, Y.; Yin, S.-B.; Li, F.-M.; Chen, P,; Chen, Y. Interfacial Engineering Enhances the Electroactivity of Frame-Like Concave RhCu Bimetallic Nanocubes for Nitrate Reduction. *Adv. Energy Mater.* **2022**, *12*, 2103916.

(16) Li, L.-X.; Sun, W.-J.; Zhang, H.-Y.; Wei, J.-L.; Wang, S.-X.; He, J.-H.; Li, N.-J.; Xu, Q.-F.; Chen, D.-Y.; Li, H.; Lu, J.-M. Highly efficient and selective nitrate electroreduction to ammonia catalyzed by molecular copper catalyst@Ti_3C_2Tx MXene. *J. Mater. Chem. A* **2021**, *9*, 21771−21778.

(17) Liu, Z.; Xing, C.; Shan, Y.; Ma, M.; Wu, S.; Ge, R.; Xue, Q.; Tian, J. Molecular Engineering of 1D Conjugated Copper Anilate Coordination Polymers for Boosting Electrocatalytic Nitrate Reduction to Ammonia. Chem. Sci. 2025, Accepted Manuscript.

(18) Yao, Y.; Wei, K.; Zhao, S.; Zhou, H.; Kui, B.; Zhu, G.; Wang, W.; Gao, P.; Ye, W. Highly Efficient Bifunctional NiFe-MOF Array Electrode for Nitrate Reduction to Ammonia and Oxygen Evolution Reactions. *ACS Sustainable Chem. Eng.* **2025**, *13*, 1245–1252.

(19) Zhou, Q.; Wang, X.; Rong, S.; Li, G.; Jiang, Q.; Pang, H.; Ma, H. Efficient Electrocatalytic Conversion of Nitrate in Water with Anderson-Type Polyoxometalate-Modified Co-MOF. *Inorg. Chem.* **2025**, *64*, 5291–5301.

(20) Su, Z.; Liu, K. K.; Xu, Y.-Q.; Yan, B.; Wang, S.; Guan, Z.-J.; Zou, Y.; Fang, Y. Charge Manipulation of Porous Coordination Cages Tunes the Efficiency and Selectivity in Electrochemical Synthesis. *Angew. Chem. Int. Ed.* **2025**, *64*, e202420945.

(21) Wang, M.; Li, S.; Gu, Y.; Xu, W.; Wang, H.; Sun, J.; Chen, S.; Tie, Z.; Zuo, J.-L.; Ma, J.; Su, J.; Jin, Z. Polynuclear Cobalt Cluster-Based Coordination Polymers for Efficient Nitrate-to-Ammonia Electroreduction. *J. Am. Chem. Soc.* **2024***, 146*, 20439–20448.

(22) Yao, Y.; Wei, X.; Zhou, H.; Wei, K.; Kui, B.; Wu, F.; Chen, L.; Wang, W.; Dai, F.; Gao, P.; Wang, N.; Ye, W. Regulating the d-Band Center of Metal–Organic Frameworks for Efficient Nitrate Reduction Reaction and Zinc-Nitrate Battery. *ACS Catal.* **2024**, *14*, 16205–16213.

(23) Huang, T.; Liang, T.; You, J.; Huo, Q.; Qi, S.; Zhao, J.; Meng, N.; Liao, J.; Shang, C.; Yang, H.; Hu, Q.; He, C. Coordination environment-tailored electronic structure of single atomic copper sites for efficient electrochemical nitrate reduction toward ammonia. *Energy Environ. Sci.* **2024**, *17*, 8360–8367.

(24) Mukherjee, N.; Adalder, A.; Barman, N.; Thapa, R.; Urkude, R.; Ghoshc, B.; Ghorai, U. K. Fe(TCNQ)2 nanorod arrays: an efficient electrocatalyst for electrochemical ammonia synthesis via the nitrate reduction reaction. *J. Mater. Chem. A* **2024**, *12*, 3352.

(25) Zhang, R..; Hong, H.; Liu, X.; Zhang, S.; Li, C.; Cui, H.; Wang, Y.; Liu, J.; Hou, Y.; Li, P.; Huang, Z.; Guo, Y.; Zhi, C. Molecular Engineering of a Metal-Organic Polymer for Enhanced Electrochemical Nitrate-to-Ammonia Conversion and Zinc Nitrate Batteries. *Angew. Chem. Int. Ed.* **2023**, *62*, 202309930.

(26) Zhang, Y.; Zheng, H.; Zhou, K.; Ye, J.; Chu, K.; Zhou, Z.; Zhang, L.; Liu, T. Conjugated Coordination Polymer as a New Platform for Efficient and Selective Electroreduction of Nitrate into Ammonia. *Adv. Mater.* **2023**, *35*, 2209855.

(27) Lv, Y.; Ke, S.-W.; Gu, Y.; Tian, B.; Tang, L.; Ran, P.; Zhao, Y.; Ma, J.; Zuo, J.-L.; Ding, M. Highly Efficient Electrochemical Nitrate Reduction to Ammonia in Strong Acid Conditions with Fe_2M-Trinuclear-Cluster Metal–Organic Frameworks. *Angew. Chem. Int. Ed.* **2023**, *62*, e202305246.

(28) Liu, P.; Yan, J.; Huang, H.; Song, W. Cu/Co bimetallic conductive MOFs: Electronic modulation for enhanced nitrate reduction to ammonia. *Chem. Eng. J.* **2023**, *466*, 143134.

(29) Padinjareveetil, A. K. K.; Perales-Rondon, J. V.; Zaoralová, D.; Otyepka, M.; Alduhaish, O.; Pumera, M. Fe-MOF Catalytic Nanoarchitectonic toward Electrochemical Ammonia Production. *ACS Appl. Mater. Interfaces* **2023**, *15*, 47294– 47306.

(30) Ma, J.; Zhang, Y.; Wang, B.; Jiang, Z.; Zhang, Q.; Zhuo, S. Interfacial Engineering of Bimetallic Ni/Co-MOFs with H-Substituted Graphdiyne for Ammonia Electrosynthesis from Nitrate. *ACS Nano* **2023**, *17*, 6687– 6697.

(31) Wang, Z.; Liu, S.; Wang, M.; Zhang, L.; Jiang, Y.; Qian, T.; Xiong, J.; Yang, C.; Yan, C. In Situ Construction of Metal–Organic Frameworks as Smart Channels for the Effective Electrocatalytic Reduction of Nitrate at Ultralow Concentrations to Ammonia. *ACS Catal.* **2023**, *13*, 9125– 9135.

(32) Ye, S.; Yang, X.; Huang, Z.; Chen, Z.; Chen, W.; Huang, T.; Ou, Z.; Xiong, W.; Li, Y.; Ren, X.; Liu J.; Zhang, Q. The Activity Origin of FeCo Prussian Blue Analogue for Ambient Electrochemical Hydrogenation of Nitrate into Ammonia in Neutral Electrolyte, *Sci. China Mater.* **2023**, *66*, 3573–3581.

(33) Zhao, S.; Zheng, Z.; Qi, L.; Xue, Y.; Li, Y. Controlled Growth of Donor–Bridge–Acceptor Interface for High-Performance Ammonia Production. *Small* **2022**, *18*, 2107136.

(34) Zhu, X.; Huang, H.; Zhang, H.; Zhang, Y.; Shi, P.; Qu, K.; Cheng, S.-B.; Wang, A.-L.; Lu, Q. Filling Mesopores of Conductive Metal−Organic Frameworks with Cu Clusters for Selective Nitrate Reduction to Ammonia. *ACS Appl. Mater. Interfaces* **2022**, *14*, 32176−32182.

(35) Guo, H.; Zhou, Y.; Chu, K.; Cao, X.; Qin, J.; Zhang, N.; Roeffaers, B. J.; Zbril, R.; Hofkens, J.; Mullen, K.; Lai, F.; Liu, T. Improved Ammonia Synthesis and Energy Output from Zinc-Nitrate Batteries by Spin-State Regulation in Perovskite Oxides. *J. Am. Chem. Soc.* **2025**, *147*, 3119−3128.

(36) Yao, Y.; Wei, X.; Zhou, H.; Wei, K.; Kui, B.; Wu, F.; Chen, L.; Wang, W.; Dai, F.; Gao, P.; Wang, N.; Ye, W. Regulating the d-Band Center of Metal–Organic Frameworks for Efficient Nitrate Reduction Reaction and Zinc-Nitrate Battery. *ACS Catal.* **2024**, *14*, 21, 16205–16213.

(37) Yan, J.; Liu, P.; Li, J.; Huang, H.; Tong, S.; Song, W. A bioinspired Fe/Mo bimetallic nitride catalyst for efficient electrochemical ammonia synthesis and Zn-nitrate battery. *Chem. Eng. J.* **2024**, *498*, 155108.

(38) Luo, W.; Guo, Z.; Ye, L.; Wu, S.; Jiang, Y.; Xu, P.; Wang, H.; Qian, J.; Zhou, X.; Tang, H.; Ge, Y.; Guan, J.; Yang, Z.; Nie, H. Electrical-Driven Directed-Evolution of Copper Nanowires Catalysts for Efficient Nitrate Reduction to Ammonia. *Small* **2024**, *20*, 2311336.

(39) Zhu, X.; Ma, C.; Wang, Y.-C.; Qu, K.; Song, L.; Wang, J.; Gong, Y.; Liu, X.; Zhang, J.; Lu, Q.; Wang, A.-L. Mott–Schottky contact synergistically boosts the electroreduction of nitrate to ammonia under low-nitrate concentration. *Energy Environ. Sci.* **2024**, *17*, 2908-2920.

(40) Gao, Y.; Wang, K.; Xu, C.; Fang, H.; Yu, H.; Zhang, H.; Li, S.; Li, C.; Huang, F. Enhanced electrocatalytic nitrate reduction through phosphorus-vacancy-mediated kinetics in heterogeneous bimetallic phosphide hollow nanotube array. *Appl. Catal. B* **2023**, *330*, 122627.

(41) Ma, J.; Zhang, Y.; Wang, B.; Jiang, Z.; Zhang, Q.; Zhuo, S. Interfacial Engineering of Bimetallic Ni/Co-MOFs with H-Substituted Graphdiyne for Ammonia Electrosynthesis from Nitrate. *ACS Nano* **2023**, *17*, 6687–6697.

(42) Zhou, J.; Wen, M.; Huang, R.; Wu, Q.; Luo, Y.; Tian, Y.; Wei, G.; Fu, Y. Regulating active hydrogen adsorbed on grain boundary defects of nano-nickel for boosting ammonia electrosynthesis from nitrate. *Energy Environ. Sci.* **2023**, *16*, 2611-2620.

(43) Chang, Z.; Meng, G.; Chen, Y.; Chen, C.; Han, S.; Wu, P.; Zhu, L.; Tian, H.; Kong, F.; Wang, M.; Cui, X.; Shi, J. Dual-Site W-O-CoP Catalysts for Active and Selective Nitrate Conversion to Ammonia in a Broad Concentration Window. *Adv. Mater.* **2023**, *35*, 2304508.

(44) Liu, Q.; Xie, L.; Liang, J.; Ren, Y.; Wang, Y.; Zhang, L.; Yue, L.; Li, T.; Luo, Y.; Li, N.; Tang, B.; Liu, Y.; Gao, S.; Alshehri, A. A.; Shakir, I.; Agboola, P. O.; Kong, Q.; Wang, Q.; Ma, D.; Sun, X. Ambient Ammonia Synthesis via Electrochemical Reduction of Nitrate Enabled by $NiCo_2O_4$ Nanowire Array. *Small*, **2022**, *18*, 2106961.

(45) Deng, Z.; Ma, C.; Li, Z.; Luo, Y.; Zhang, L.; Sun, S.; Liu, Q.; Du, J.; Lu, Q.; Zheng, B.; Sun, X. High-Efficiency Electrochemical Nitrate Reduction to Ammonia on a Co_3O_4 Nanoarray Catalyst with Cobalt Vacancies. *ACS Appl. Mater. Interfaces* **2022**, *14*, 46595–46602.

(46) Xie, L.; Sun, S.; Hu, L.; Chen, J.; Li, J.; Ouyang, L.; Luo, Y.; Alshehri, A. A.; Kong, Q.; Liu, Q.; Sun, X. In Situ Derived Co_2B Nanosheet Array: A High-Efficiency Electrocatalyst for Ambient Ammonia Synthesis via Nitrate Reduction. *ACS Appl. Mater. Interfaces* **2022**, *14*, 49650–49657.

(47) Zhang, R.; Guo, Y.; Zhang, S.; Chen, D.; Zhao, Y.; Huang, Z.; Ma, L.; Li, P.; Yang, Q.; Liang, G.; Zhi, C. Efficient Ammonia Electrosynthesis and Energy Conversion through a Zn-Nitrate Battery by Iron Doping Engineered Nickel Phosphide Catalyst. *Adv. Energy Mater.* **2022**, *12*, 2103872.
